# A conserved element in the first intron of *Cd4* has a lineage specific, TCR signal-responsive, canonical enhancer function that matches the timing of cell surface CD4 upregulation required to prevent lineage choice error

**DOI:** 10.3389/fimmu.2024.1469402

**Published:** 2025-01-15

**Authors:** Gregory A. Swan, Chika Fujii, Mia E. Guzynski, Sheridan M. Page, Isabelle V. Meyers, Yordan P. Penev, Sejiro Littleton, Adinda Azzahra, Christine Richardson, Sophia D. Sarafova

**Affiliations:** ^1^ Integrative Immunobiology Department, Duke University, Durham, NC, United States; ^2^ Biology Department, Davidson College, Davidson, NC, United States; ^3^ Department of Biological Sciences, University of North Carolina-Charlotte, Charlotte, NC, United States

**Keywords:** CD4, T-cell development, transcriptional regulation, helper T-cell lineage, enhancer function

## Abstract

**Introduction:**

The regulation of *Cd4* expression during T-cell development and immune responses is essential for proper lineage commitment and function in the periphery. However, the mechanisms of genetic and epigenetic regulation are complex, and their interplay not entirely understood. Previously, we demonstrated the need for CD4 upregulation during positive selection to ensure faithful commitment of MHC-II-restricted T cells to the CD4 lineage. In this study, we investigate whether a conserved region, here called NCE, that is proximal to the Cd4 silencer and contains E4m has the required developmental-stage-specific canonical enhancer function and TCR responsiveness to mediate the CD4 upregulation required to prevent lineage errors.

**Methods:**

To investigate the role of NCE, transient transfection of reporter plasmids was performed in thymoma cell lines arrested at the double-positive (DP, CD4^+^CD8^+^) and intermediate (INT, CD4^+^CD8^lo^) stages of development. CRISPR/Cas9-mediated deletion of the coreNCE/E4m region was carried out in these cell lines to assess its impact on CD4 surface expression, re-expression rates, and TCR signaling responsiveness. To avoid developmental alterations from direct manipulation of the endogenous *Cd4* locus *in vivo*, BAC-transgenic reporter mice were generated with the locus modified to express EGFP in the presence or absence of NCE. EGFP mRNA levels were measured via RT-qPCR, and EGFP fluorescence was analyzed in post-selection thymocytes.

**Results:**

Our *in vitro* experiments demonstrate that NCE by itself can function as an enhancer at the INT, but not the DP stage of development. Furthermore, CRISPR/Cas9-mediated deletion of coreNCE/E4m resulted in reduced CD4 surface levels, slower re-expression rates, and reduced TCR signaling responsiveness in INT cells, but not in DP cells. *In vivo*, NCE-sufficient transgenic mice exhibited upregulation of Cd4 reporter EGFP mRNA levels at the INT stage and a corresponding upregulation of EGFP fluorescence, whereas NCE-deficient mice showed a significant loss of *Cd4* reporter EGFP mRNA and no detectable EGFP production in any post-selection thymocytes.

**Discussion:**

This study demonstrates that the canonical enhancer function of coreNCE/E4m is essential for CD4 upregulation following positive selection. The NCE region, with its developmental-stage-specific activity and its known epigenetic regulatory capabilities, ensures faithful lineage commitment to the CD4 lineage.

## Introduction

The developmental decision to differentiate into a helper or cytotoxic T-cell begins with positive selection of CD4^+^CD8^+^ (DP) cells in the thymus, and the subsequent kinetics of CD4 and CD8 coreceptor cell-surface expression play a central role in the functional lineage decision (1, 2). The role of CD8 and how its kinetics of expression is regulated is well understood (2–5); however, the role of CD4 and the regulation of its kinetics of expression in this lineage commitment process remain not fully characterized.

The CD4 glycoprotein encoded by the *Cd4* gene, functions as a coreceptor for the T-cell receptor (TCR) in two ways—one involves binding an invariant region in the alpha2 chain of MHC-II, thus stabilizing the MHC-TCR interaction, and the other involves recruiting the tyrosine kinase Lck to the TCR for initiation of the signal (6, 7). Missing either the binding site for Lck on CD4 and CD8 or the extracellular domain specific for the corresponding MHC renders the TCR independent of MHC and T cells are no longer MHC restricted (8–10), impacting both positive selection and lineage commitment.

Simply expressing CD4 on the cell surface is not sufficient to guarantee the matching between MHC class, coreceptor, and lineage function. Changing the kinetics of CD4 cell surface expression by placing the *Cd4* gene under the control of various *Cd8* regulatory elements reveal that the kinetics of CD4 expression, specifically its upregulation immediately following positive selection at the CD4^+^CD8^lo^ intermediate (INT) stage, rather than its presence or absolute amount, is essential for correct lineage decision during T-cell development (1, 11, 12). If CD4 levels fail to increase after positive selection to ensure the continued interaction of lower-affinity MHC-II-specific TCRs, CD8^+^ MHC-II-specific cytotoxic T cells develop with a clear mismatch between their functional lineage and MHC restriction of their TCR (11). Reciprocal experiments in which the *Cd8* gene is placed under *Cd4* regulation, such that CD8 levels are forced to increase and persist after positive selection, also produces a mismatch between the functional lineage and TCR specificity (13). Combining these two features to generate the flip/flop mouse model solidifies the notion that the kinetics of expression, not the strength of the signal or the identity of the coreceptor, is essential for lineage commitment (1).

**Figure 1:**
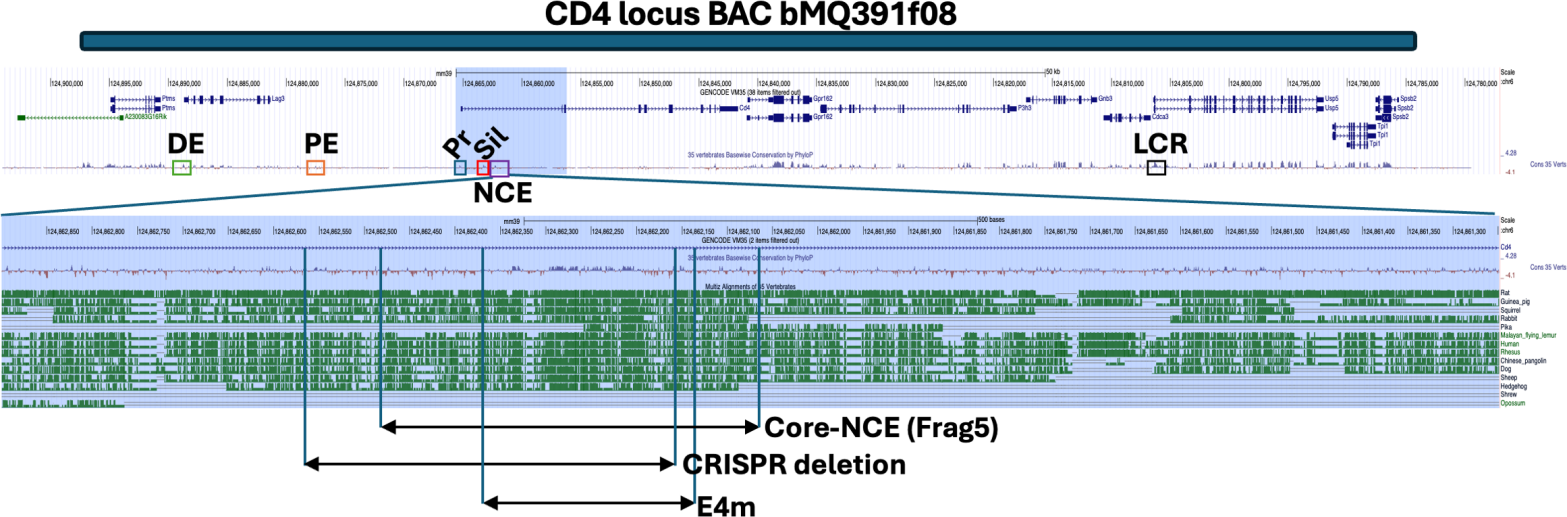
A region of high homology across vertebrate species in the *Cd4* gene. A UCSC’s genome browser map (http://genome.ucsc.edu) (45) of the *Cd4* locus on chromosome 6 with aligned BAC bMQ391f08 used in this study (blue bar) and the known regulatory elements of *Cd4* are indicated: DE, distal enhancer (green box); PE, proximal enhancer (orange box); Pr, promoter (blue box); Sil, silencer (red box); NCE, novel cis-acting element downstream of Sil; the conserved region of interest (purple box); and LCR, locus control region (black box). Cons: conserved base pairs identified by *PhyloP* a part of the Phylogenic Analysis with Space/Time models (PHAST) package. The first intron of *Cd4* is highlighted in blue and the NCE region expanded to indicate the location of the coreNCE and the CRISPR deletion in this study relative to the published epigenetic regulatory element E4m.

The regulatory elements that generate the dynamic pattern of CD4 expression during positive selection and lineage choice remain under investigation. Initial transgene experiments involving a 100 kb cosmid of the *Cd4* locus faithfully recapitulated the expression pattern of CD4 (14) and helped identify a promoter, a silencer, a locus control region, and at least two enhancers (15–17) (**Figure 1**). However, a transgene with only these elements was not able to direct the expression of a reporter at all stages of T-cell development (11, 18, 19). In fact, most *Cd4* transgenic models, especially the ones using heterologous promoters and enhancers, fail to recapitulate the correct timing and level of CD4 expression and generate varying degrees of lineage choice errors unless the entire *Cd4* locus is included (10, 11, 18, 20). These findings suggest that the known regulatory elements are not sufficient to establish the complicated pattern of developmental regulation of CD4, and we think that an additional positive regulatory element should exist and should be able to function as a TCR-signal-responsive enhancer during positive selection.

Further supporting the likelihood of an additional positive regulatory element, when transcription factor occupancy of the known promoter and enhancers is monitored during development, usage of the proximal and distal enhancers diminishes with increasing levels of maturation, despite increasing cell surface levels of CD4 (21). Also, when the *Cd4* silencer is deleted together with an additional 1.1 kb downstream sequence, CD4 expression is dysregulated such that in addition to incorrectly appearing on CD8^+^ cytotoxic-lineage cells, it has decreased expression in CD4 single positive (SP) helper-lineage cells, which is unexpected when removing a negative cis-acting regulatory element (22, 23). Some of the cytotoxic-lineage cells in these mice continue to develop in the absence of MHC-I, indicating that they are MHC-II-specific cells that have made an error in their lineage choice (22). A more targeted deletion of the silencer leaves CD4 expression levels on CD4SP cells unaffected and does not generate lineage decision errors (24), supporting the notion that a positive regulatory element is present downstream of the silencer in the first intron of *Cd4* and needs to be identified and characterized to gain a full understanding of the regulation of *Cd4* expression as well as the developmental and functional consequences of dysregulation.

While we were exploring the area downstream of the *Cd4* silencer we call NCE (25), other labs investigated the same region for epigenetic regulation of CD4 (26–29). In the absence of the silencer together with 1 kb of immediately downstream sequence, CD4 expression is unstable (29) and resulted in some lineage redirection of MHC-II restricted thymocytes to the killer T-cell lineage via a DNA demethylation mechanism (26, 29). A more precise deletion within the silencer downstream region, called E4m resulted in hypermethylation of the *Cd4* locus, which is exacerbated when the proximal enhancer (PE or E4p) is also deleted leading to models of regulatory element collaboration (26, 27). This collaboration is further supported by the finding that in the absence of the proximal enhancer and the silencer, E4m supported only very low levels of CD4 expression in the thymus (26). Finally, several transcription factors involved in chromatin remodeling have been described as essential (Bcl11b) or suppressive (Runx1, Runx3, and Th-Pok) of E4m function (26).

Given that all of these studies are done in mice, where the physiological functions of regulatory elements are best studied, there is no doubt that the epigenetic capabilities of E4m are an essential component of its function, however under these circumstances it is difficult to separate the classical transcriptional enhancer function from the epigenetic function of the element. To our knowledge there has been no direct demonstration of position and orientation independent transcriptional regulation that directly affects the rate of *Cd4* gene expression in a TCR signal-responsive and developmental stage-specific manner. Furthermore, by the nature of the *in vivo* studies of E4m, the deletion of the various *Cd4* regulatory elements alter the endogenous *Cd4* locus and as a result T- cell development. Therefore, it would be beneficial to confirm that the region containing E4m generates the same expression pattern when regulating a reporter gene during normal T cell development in a mouse with an unaltered endogenous *Cd4* locus.

In this paper we describe the identification and characterization of NCE that enhances CD4 expression after positive selection in the thymus and contains the E4m epigenetic control region (29). We demonstrate that NCE functions as a TCR-responsive, developmental stage-specific canonical enhancer *in vitro* and is indispensable for CD4 expression *in vivo*. In transient transfections with reporter plasmids, NCE enhanced *Cd4* promoter function in a cell line arrested at the INT stage of development but had no effect on a cell line arrested at the DP stage of development. Furthermore, CRISPR deletion of the coreNCE conserved region, which contains E4m (**Figure 1**), in the same cell lines resulted in a decrease of CD4 cell surface levels in INT stage cells but not in DP cells. In addition, stripping of cell surface CD4 with pronase and following the kinetics of re-emergence of newly synthesized CD4 over time revealed a decreased rate of expression in the absence of coreNCE/E4m in INT but not DP stage cells. Finally, stimulated coreNCE/E4m-deficient INT cells upregulated their CD4 levels significantly less than coreNCE/E4m-sufficient INT cells, while DP cells were unaffected, suggesting that the highly conserved region functions as a TCR-responsive, developmental-stage-specific enhancer *in vitro*. Investigation of NCE *in vivo* in BAC-transgenic mice that express an EGFP reporter instead of CD4 from the transgenic *Cd4* locus revealed that the deletion of NCE resulted in EGFP expression at the DP stage that decreased by 80% after positive selection, as measured qPCR and became undetectable by flow cytometry, consistent with the behavior of the E4m-deleted endogenous *Cd4* studies (26). Therefore, for the first time, we demonstrate that the conserved region in the first intron of *Cd4*, immediately downstream of the silencer, has an important canonical enhancer function at the INT stage of development, best demonstrated *in vitro*. Its enhancer function also contributes to the upregulation of CD4 expression after positive selection *in vivo* and is required for maintenance of CD4 expression, presumably via its demethylation capabilities.

## Results

### A highly conserved region in the first intron of *Cd4* has the properties of an enhancer

To identify new *cis* regions that potentially regulate *Cd4* expression, we started by identifying highly conserved sequences in untranslated portions of the *Cd4* gene. An alignment of *Cd4* from multiple vertebrate species revealed a highly conserved region of 150 bp approximately 1 kb downstream from the silencer in the first intron (**Figure 1**). This area exhibits many of the characteristics of an enhancer responsible for CD4 upregulation during positive selection, including decreased CD4 cell surface expression on CD4SP cells and lineage choice errors in its absence (22, 23). Analysis of the area by ATAC-seq and genome-wide ChIP-seq has shown that the region is accessible to TFs and is enriched for p300 occupancy in CD4SP cells, both of which are indicative of enhancer function (21, 30). Given these clues, we cloned a 1.7 kb piece of DNA downstream of the *Cd4* silencer we call NCE, which contains the highly conserved 150 bp region also contained in coreNCE/E4m (**Figure 1**) and began investigating whether it can provide canonical enhancer function *in vitro*.

### NCE functions as a TCR-responsive, developmental stage-specific canonical enhancer *in vitro*

**Figure 2:**
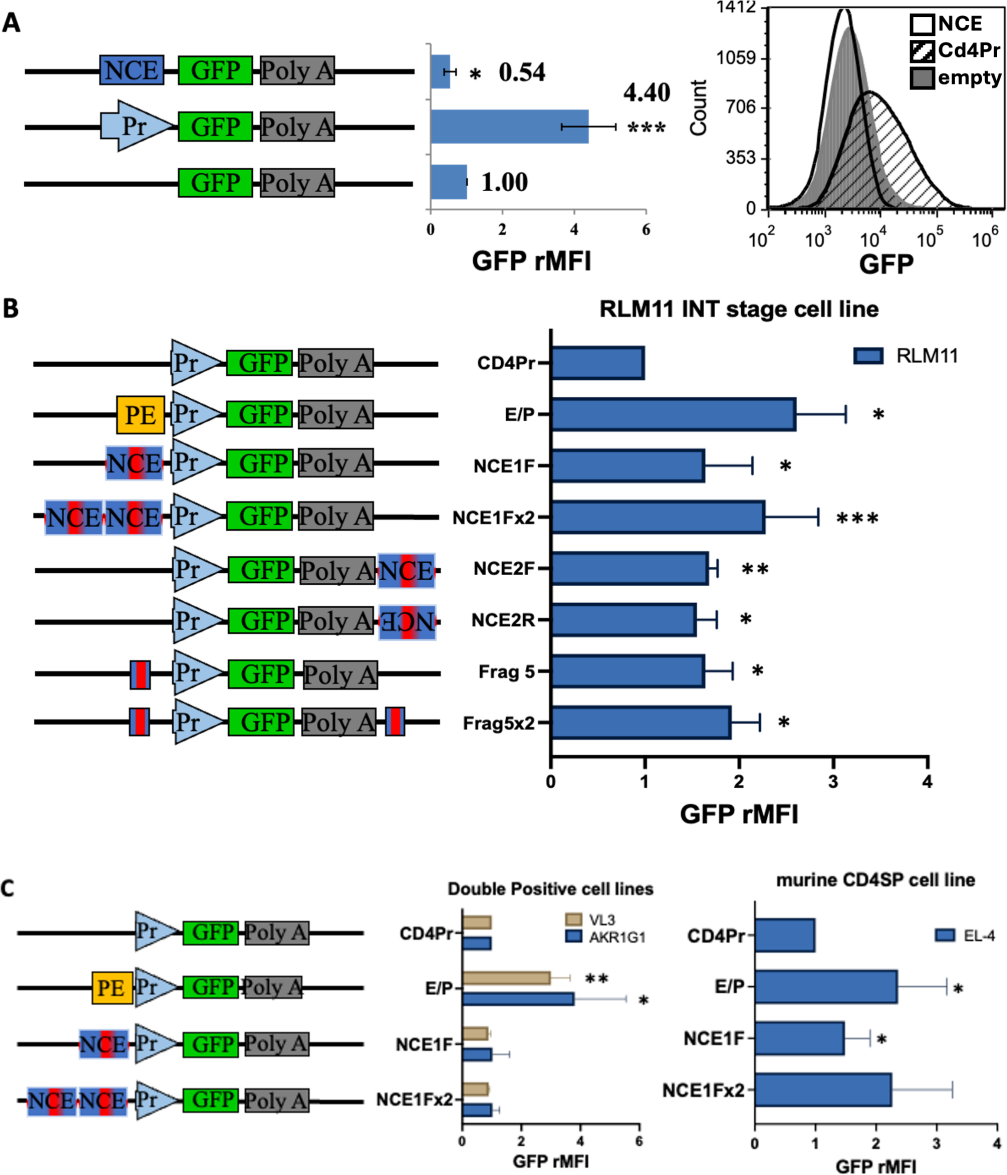
NCE has intrinsic enhancer function at the INT but not the DP stage of development *in vitro*. Schematic representations of the constructs tested are shown on the left of each panel. Pr, PE, and NCE are the same as in **Figure 1**; red bar within NCE = 150 bp highly conserved sequence contained in the 414bp coreNCE(Frag 5) (**Figure 1**) Bar graphs represent the relative EGFP MFI of successfully transfected cells, identified as in **Supplementary Figure S1**, after transient co-transfections of each construct with a transfection control plasmid, as measured using an AccuriC6 flow cytometer and Cflow Plus or FCS Express Software. **(A)** Transient transfections of RLM11 cells to test NCE for promoter function. Measurements were normalized to those of cells transfected with empty vector pd2EGFP-1. The histogram shows the EGFP fluorescence distribution of cells with the empty vector (grey fill), NCE construct (black line), and *Cd4* promoter construct (black hashed fill) for a single trial. **(B)** Transient transfections testing for NCE enhancer function in RLM11 INT stage thymoma. **(C)** DP stage thymomas AKR1G1 and VL3, and the CD4SP cell line EL-4. Bar graphs are normalized to the construct containing the *Cd4* promoter alone (Pr). Significant enhancement of *Cd4* promoter-driven EGFP expression was determined using a two-tailed t test comparison to a standard value; * indicates p <0.05, ** indicates p <0.01, and *** indicates p <0.005. n = at least 3 and up to 10 repeats. Error bars represent the standard error of the mean.

Many genes with complex regulation have multiple promoters and enhancers (31–33), including the human *CD4* gene, which has a second promotor in its first intron (34, 35). As NCE is in the first intron of the murine *Cd4* gene and the translation start codon is in exon 2, we reasoned that alternative promoter function is possible for NCE that could alter the timing or the level of expression of *Cd4* without any changes in the CD4 protein sequence. Thus, we investigated whether NCE has promoter function and could serve as an alternative or developmental stage specific promoter by generating reporter constructs that place EGFP under the control of NCE alone, the *Cd4* promoter alone (Pr), or the *Cd4* promoter with the *Cd4* proximal enhancer (PE) (**Figure 2**, **Supplementary Figure S1**). To achieve maximum sensitivity to small changes in expression in the transient transfection assay, we used EGFP, which has a proteasome degradation signal that shortens its half-life to two hours (36). As we expected NCE to function at the intermediate (INT) stage of development, we transfected RLM11 cells, which have terminated CD8 expression, express CD4, but have not yet expressed the CD4 lineage master regulator Th-Pok (ThPok^-^ CD4^+^CD8^-^) and therefore best resemble positively selected INT stage thymocytes that have not committed to the CD4 helper lineage (37). To identify transfected cells independently of the reporter construct activity, we co-transfected them with a CMV-driven *Cd8* control vector. Successfully transfected RLM11 cells expressed high levels of CD8. We measured the reporter EGFP mean fluorescence intensity (MFI) of the CD8^hi^ cells relative to that of the empty vector (**Figure 2A**, **Supplementary Figure S1**). We found that the *Cd4* promoter produced approximately fourfold more EGFP than did the empty vector, and the addition of the proximal enhancer further doubled the expression level, indicating that the assay is sensitive enough to detect changes in the level of expression driven by known *Cd4* regulatory elements (**Supplementary Figure S1**). In contrast, NCE alone did not promote any EGFP expression and even exhibited some statistically significant suppressive effect (**Figure 2A**), leading us to the conclusion that NCE cannot function as a promoter *in vitro*.

As enhancers are known to operate in a position- and orientation-independent manner (38), we investigated the potential enhancer function of NCE by generating additional constructs in which we placed NCE in different positions and orientations relative to the *Cd4* promoter (**Figure 2B**). Given that the upregulation of CD4 from the signaled DP to the INT stage of development is approximately 1.5- to 2-fold (11), we expected only a modest enhancer function in RLM11 cells. We observed a consistent and statistically significant 1.5-fold increase in EGFP expression in all NCE positions and orientations tested (**Figure 2B**, constructs NCE1F, NCE2F, NCE2R). The NCE enhancer function was additive, as we consistently observed higher EGFP fluorescence in the presence of two NCE elements compared to one NCE element (**Figure 2B**, NCE1Fx2 vs NCE1F), indicating that NCE by itself can enhance *Cd4* promoter activity in cells immortalized at the INT stage.

To determine whether the canonical enhancer capability of NCE resides in the highly conserved region, coreNCE/E4m, we generated two additional constructs with one (Frag5) or two (Frag5x2) copies of the coreNCE/E4m and compared their activity to that of the corresponding NCE1F and NCE1Fx2 (**Figure 2B**). In both cases, coreNCE/E4m performed as well or better than NCE in terms of enhancer function in RLM11 cells, although the difference was not statistically significant (**Figure 2B**), indicating that coreNCE/E4m is sufficient to support canonical enhancer function *in vitro.*

We next sought to elucidate the developmental stage specificity of NCE. We repeated the transient transfection experiments in a variety of cell lines immortalized at different stages of T-cell development. NCE was not able to enhance *Cd4* promoter function in the DP thymoma cell lines AKR1G1 or VL3-3M2 (**Figure 2C**), while the proximal enhancer (PE) functioned significantly better in these cell lines than in RLM11, consistent with previous observations that the proximal enhancer ability decreases with increasing maturation (21, 27). At the CD4SP stage, we found barely significant NCE enhancer function in the murine EL-4 CD4SP lymphoma cell line with the NCE1F, but not with the NCE1Fx2, even though the proximal enhancer was still functional (**Figure 2C**), indicating that NCE’s canonical enhancer function may diminish in mature CD4SP T cells. As previously reported (29), there was no detectable NCE enhancer function in the human Jurkat CD4SP cell line, even though murine PE enhances murine *Cd4* promoter function in this cell line as well (**Supplementary Figure S2**).

**Figure 3:**
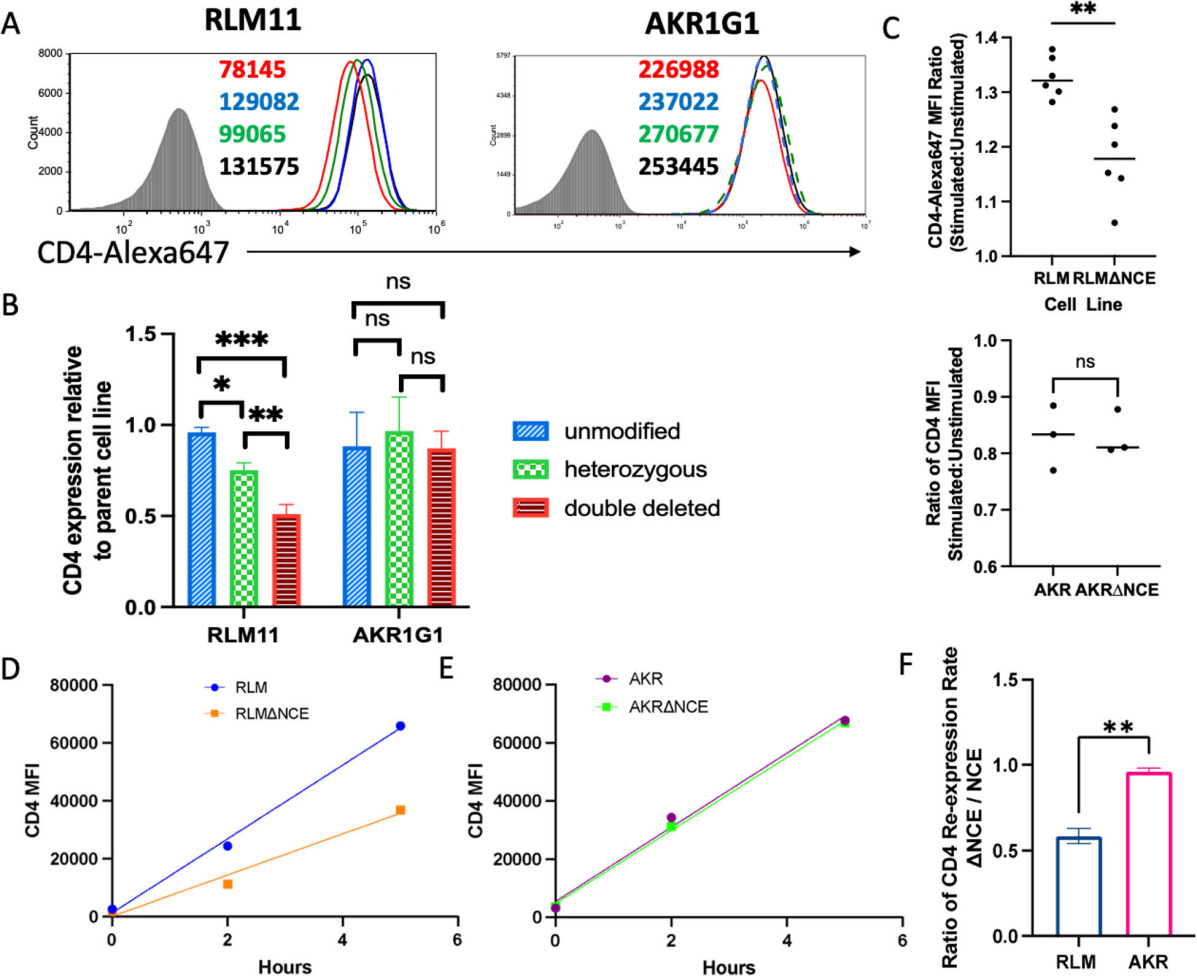
Deletion of coreNCE/E4m reduces CD4 cell surface expression in a developmental stage-specific manner and significantly reduces TCR responsiveness *in vitro.* **(A)** CD4 cell surface expression levels on RLM11 and AKR1G1 cells with CRISPR-Cas9 deletion of the coreNCE/E4m on none (blue), one (green), or both (red) of the alleles of *Cd4* compared to the corresponding parental cell lines (black). The numbers in the histograms represent the CD4 MFI of individual clones. Shaded histogram is the isotype control stain. **(B)** The bar graph represents the relative CD4 expression normalized to that of the parental cell line; n = at least 2 and up to 5 different clones. The Turkey-Kramer HSD multiple comparison test was used to show that coreNCE/E4m- unmodified, heterozygous, and double-deleted clones are significantly different from each other in RLM11, but not AKR1G1 cells ns = not significant; *p<0.05; **p<0.01; ***p<0.001. **(C)** The ratio of the CD4 MFI of ConA-stimulated (CD69^+^) to that in unstimulated (CD69^-^) RLM11 and coreNCE/E4m-deleted RLM11 (ΔNCE) cells as well as in ConA-stimulated (CD5^+^) to that in unstimulated (CD5^-^) AKR1G1 and coreNCE/E4m-deleted AKR1G1 cells as a measure of responsiveness to TCR stimulation. A two-tailed t test was used to determine whether this ratio was significantly different between NCE-sufficient and NCE-deficient cells.n = 6 for RLM11 and n = 3 for AKR1G1; **p<0.01; ns = not significant **(D–F)** Kinetics of CD4 re-expression in unmodified and coreNCE/E4m-deleted RLM11 **(D)** and AKR1G1 **(E)** cells stripped of cell surface CD4 using pronase. Linear regression was performed to determine the rate of CD4 re-expression, and the ratio of CD4 expression rates (ΔNCE: NCE) was calculated for each cell line **(F)**. A two-tailed t test was performed to determine whether this ratio was significantly different between RLM and AKR cells. The error bars represent +/- 1 standard deviation. n = 3; **p<0.01. *p<0.05; ***p<0.001. ns = not significant.

As we had demonstrated canonical transcriptional enhancer function using transient transfections, we next wanted to determine whether coreNCE/E4m is necessary for enhanced *Cd4* expression in its native genomic context. We deleted the coreNCE/E4m in the *Cd4* locus on mouse chromosome 6 in RLM11 and AKR1G1 cells using a CRISPR/Cas9 approach and investigated the effect of the deletion on CD4 cell surface expression. Clones in which the deletion was unsuccessful did not have a significant change in their cell surface CD4 levels, indicating that there were no off-target effects that influenced *Cd4* expression (**Figures 3A, B**, blue histograms and bars). Successful deletion of coreNCE/E4m in RLM11 cells on one *Cd4* allele decreased CD4 cell surface levels by 25%, and deletion on both *Cd4* alleles resulted in a 50% reduction relative to that of the parental cell line (**Figures 3A, B**, green and red histograms and bars). In contrast, coreNCE/E4m deletion on one or both *Cd4* alleles in AKR1G1 cells did not significantly alter CD4 cell surface expression (**Figures 3A, B**), suggesting that coreNCE/E4m is required for full expression of *Cd4* at the INT but not the DP stage of development. As we expected coreNCE/E4m to be responsive to TCR signaling, we compared the ability of NCE-sufficient and NCE-deficient RLM11 cells to upregulate CD4 in response to TCR stimulation. When TCRs were crosslinked with concanavalin A, coreNCE/E4m-sufficient RLM11 cells upregulated CD4 expression on the cell surface significantly better than coreNCE/E4m-deficient RLM11 cells (**Figure 3C**). AKR1G1 DP cells exhibited reduced CD4 surface levels when stimulated with concanavalin A regardless of the presence or absence of coreNCE/E4m (**Figure 3C**).

As the surface level of CD4 represents a steady-state average of CD4 production, we wanted to compare the rate of CD4 expression in the presence vs absence of coreNCE/E4m. For that purpose, we removed preexisting CD4 from the cell surface using a previously established pronase stripping protocol (39) and monitored CD4 re-expression over time. The rate of re-expression in coreNCE/E4m-deficient RLM11 cells was half that in coreNCE/E4m-sufficient RLM11 cells (**Figure 3D**), indicating that the regulatory element is required for optimal CD4 expression, most likely as the result of a reduced transcription rate, as it is spliced out of the final mRNA product. Deletion of coreNCE/E4m did not significantly affect the rate of CD4 re-expression in AKR1G1 cells, confirming the stage specificity of the enhancer (**Figures 3E, F**).

Finally, as there is a Th-Pok binding site in coreNCE/E4m and it has been reported that Th-Pok can repress E4m function in post-selection thymocytes (26), we checked whether the expression of Th-Pok in INT stage RLM11 cells, which lack it, would alter the levels of CD4 in an NCE-dependent manner. We observed a small (~12%) but significant decrease of CD4 levels regardless of the presence or absence of coreNCE/E4m in Th-Pok transfected RLM11 (**Supplementary Figure S3**), indicating that NCE is not strictly required for Th-Pok to exert its effect in this INT stage representative cell line.

Taken together these data led us to conclude that coreNCE/E4m behaves as a TCR-responsive, developmental stage specific enhancer *in vitro* and proceeded with the investigation of its properties *in vivo.*

### NCE is required for *Cd4* expression *in vivo* in post selection thymocytes

**Figure 4:**
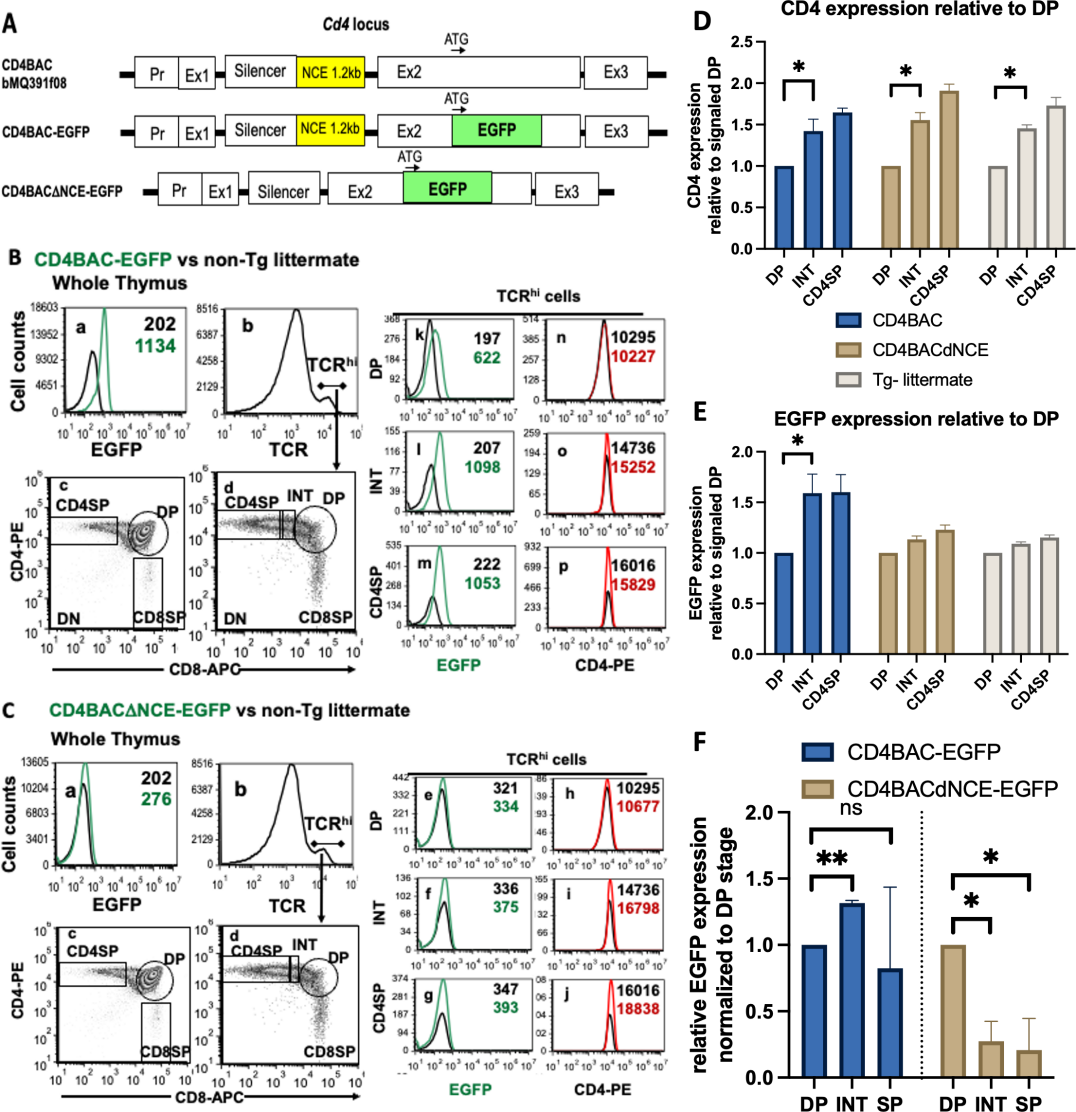
Upregulation of the CD4 reporter EGFP with increasing maturity in post-selection thymocytes requires NCE *in vivo.* **(A)** Schematic representation of the BACs used to generate CD4BAC-EGFP and CD4BACΔNCE-EGFP mice. Whole thymus staining of CD4BAC-EGFP **(B)** and CD4BACΔNCE-EGFP **(C)** thymocytes (panels a-c). Gating strategy for TCR^hi^ cells (panels b,d); EGFP (panels k-m & e-g) and CD4 (panels n-o & h-j) cell surface levels on postselection (TCR^hi^) thymocytes identified by immunostaining with anti-CD4-PE, anti-CD8-APC, and anti-TCRβ-bio (visualized with streptavidin-PerCP-Cy5.5) antibodies and flow cytometry. The black histograms represent non-transgenic littermate control cells stained with the same antibodies. **(D)** Endogenous CD4 and **(E)** transgenic EGFP levels relative to the signaled DP population in CD4BAC-EGFP mice (blue), CD4BACΔNCE-EGFP mice (beige), and nontransgenic littermates (gray) The error bars represent +/- 1 standard deviation. n = at least 3 and up to 5 mice per strain; *p<0.025. **(F)** EGFP mRNA expression on sorted thymocyte populations relative to endogenous *Cd4* of the same sample and normalized to the DP level of expression in the same mouse. The error bars represent +/- 1 standard deviation. n = 3 mice per strain; *p<0.016, **p<0.0032. ns = not significant.

Once we understood the intrinsic enhancer ability of NCE, we wished to study it in the more complex *in vivo* setting where it can exhibit its epigenetic effects in combination with its enhancer function. We used BAC recombineering on a 134 kb BAC that contains 125 kb of genomic DNA from the *Cd4* locus, which includes all known enhancers, silencers, and locus control regions (**Supplementary Figure S4**), to generate two constructs, one with NCE intact and the other with a 1.25 kb of NCE deleted (**Supplementary Figure S5**). In addition, we replaced 80 bp of exon 2 immediately after the start codon with EGFP, which contains its own stop codon (**Supplementary Figure S6**), ensuring that the engineered *Cd4* locus will express EGFP instead of CD4 in the presence (CD4BAC-EGFP) or absence (CD4BACΔNCE-EGFP) of NCE (**Figure 4A**). We verified the BAC constructs by restriction enzyme digestion and pulsed field gel electrophoresis (**Supplementary Figure S7**) and generated transgenic mice (**Supplementary Figure S8**). CD4BACΔNCE-EGFP mice were bred to homozygosity and have two copies of the transgene, while CD4BAC-EGFP mice have six copies of the transgene when homozygous (**Supplementary Figure S9**).

To compare EGFP expression levels at different stages of development, we started with flow cytometry analysis of thymocytes. EGFP expression was low but reliably detectable in the CD4BAC-EGFP mice and closely followed the CD4 expression patterns at all stages of development (**Figure 4B**). Looking at the TCR^hi^ thymocytes, we observed that EGFP exhibited the same pattern of upregulation as the characteristic CD4 upregulation in cells progressing from the signaled DP to the INT stages of development and persisted through the CD4SP stage (**Figure 4B**). This upregulation was significant both for CD4 expressed from its endogenous locus and for the CD4 reporter EGFP expressed from the CD4BAC-EGFP transgene (**Figures 4D, E**). The same pattern of *Cd4* reporter EGFP expression was observed on the mRNA level with significant upregulation of EGFP mRNA from DP to INT stage as measured by RT-qPCR on sorted populations (**Figure 4F**), consistent with the transcriptional enhancer function observed *in vitro*. In the absence of NCE, EGFP expression in CD4BACΔNCE-EGFP mice was at the limit of detection, due to the lower transgene copy number and the short half-life of EGFP (**Figure 4C**). Nevertheless, we quantified the changes in both CD4 and EGFP levels relative to those in signaled DP cells and noticed that CD4BACΔNCE-EGFP mice had a similar increase in endogenous CD4 levels as did CD4BAC-EGFP mice but did not experience a measurable change in EGFP (**Figures 4D, E**). We confirmed that there was EGFP mRNA expression in the CD4BACΔNCE-EGFP mice, however unlike the CD4BAC-EGFP mice this expression was highest at the DP stage and decreased by 80% in the INT and DP stages, indicating a requirement for NCE for *Cd4* expression in post-selection thymocytes.

**Figure 5:**
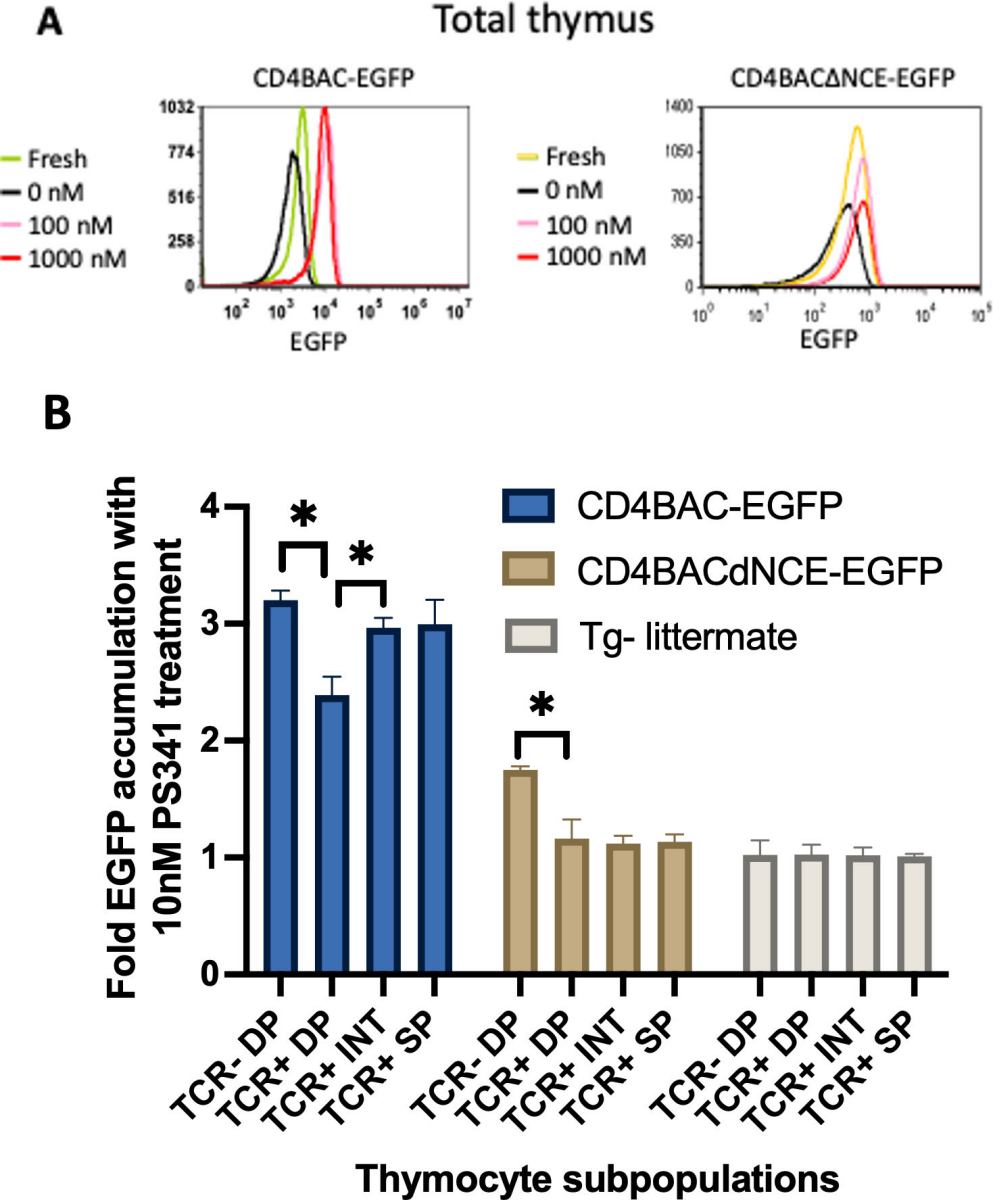
NCE is required for EGFP and mRNA expression in the CD4 reporter in post-selection thymocytes *in vivo.* **(A)** Histograms depicting the mean EGFP fluorescence intensity in the whole thymus after 24 hours of treatment with the indicated amounts of the proteasome inhibitor PS341 in CD4BAC-EGFP and CD4BACΔNCE-EGFP thymocytes. **(B)** Average fold EGFP accumulation at the indicated developmental stages, identified as in **Figure 4**, after 24 hours of 10nM PS341 treatment, calculated as the ratio of EGFP MFI of treated vs untreated cells from the same population of each mouse. CD4BAC-EGFP mice (blue bars), CD4BACΔNCE-EGFP mice (beige bars), and nontransgenic littermates (gray bars). The error bars represent +/- 1 standard deviation. n = 3 mice per strain; *p<0.05.

Finally, we wanted to investigate more closely the *in vivo* rate of *Cd4* reporter EGFP transcription at all stages of development, similarly to the *in vitro* pronase experiments described above. To that end, we took advantage of the proteasome degradation signal on EGFP that we used for both the *in vitro* reporter constructs and for the construction of the BAC transgenes. We blocked proteasome degradation using the inhibitor PS341 and monitored EGFP accumulation, which would only happen if there were active transcription of the transgene, while no accumulation would indicate simply stabilizing the existing EGFP and no new production Blocking the proteasome increased the amount of EGFP relative to untreated cells in both strains of BAC-transgenic mice, confirming that both mouse strains transcribe and translate the transgene (**Figure 5A**). However, the fold change in accumulation was not the same at different stages of development. Pre-selection thymocytes (TCR^-/lo^ DP) from both CD4BAC-EGFP and CD4BACΔNCE-EGFP thymi exhibited significant accumulation of EGFP after 24 hours of PS341 treatment compared to that in untreated cells from the same mouse. The fold change in accumulation was smaller in the CD4BACΔNCE-EGFP mice than in the CD4BAC-EGFP mice (**Figure 5B**), consistent with the lower transgene copy number. Post-selection thymocytes continued to significantly accumulate EGFP in CD4BAC-EGFP mice but not in CD4BACΔNCE-EGFP mice. In fact, there was no significant EGFP accumulation in any of the TCR^+^ cells from CD4BACΔNCE-EGFP mice (**Figure 5B**), indicating that the pre-existing EGFP before PS341 treatment was stabilized but there was little if any new production of EGFP.

## Discussion

In this study, we directly demonstrated the canonical transcriptional enhancer function of the E4m-containing regulatory element NCE (**Figure 1**) as a critical regulatory element responsible for the upregulation of CD4 in response to the positive selection signal via the TCR *in vitro.* This function is developmental stage specific, best observed in INT stage cells, TCR-responsive, and independent of Th-Pok (**Figures 2**, **3**, **Supplementary Figure S3**). We use EGFP-expressing BAC reporter transgenes of the entire *Cd4* locus, to monitor expression in mice with intact endogenous *Cd4* and therefore normal T cell development. We show that NCE is necessary to upregulate expression at the INT stage and maintain it at CD4SP stage of development, consistent with studies that have a direct deletion of E4m in the endogenous *Cd4* locus (**Figures 4**, **5**).

We demonstrated that NCE by itself can enhance *Cd4* promoter-driven transcription directly *in vitro* (**Figure 2**), indicating that its enhancer function is intrinsic and does not require other enhancers from the *Cd4* locus for this function, although it may cooperate with them. To our knowledge this is the first time the position- and orientation-independent enhancer function of the element has been shown directly. Enhancer function was observed only in cells immortalized at post-selection stages of development (INT and murine CD4SP), and not cells immortalized at the DP stage, indicating that NCE is a developmental-stage specific enhancer (**Figure 2**). Working in cell lines for the transient transfection experiments has the advantage that the plasmids used are free of epigenetic marks and we can observe directly the canonical enhancer features of NCE. The drawback is that the immortalization process could alter the gene expression in these cells and influence the experimental outcomes. To account for this possibility, we investigated two DP stage and two CD4SP stage cell lines, however to our knowledge RLM11 is the only cell line characterized by the absence of both Th-Pok and CD8 expression (37). Although we cannot rule out the possibility that some RLM11-specific features not related to the developmental stage are responsible for the NCE enhancer activity in those cells, we think that combined with the *in vivo* data the important features that are consistent across all experiments are the properties of the INT stage of development in relation to the presence or absence of coreNCE/E4m.

The absence of Th-Pok in the RLM11 cells and the presence of a Th-Pok consensus sequence binding site in coreNCE/E4m indicated that the enhancer function observed must be independent of Th-Pok. This is consistent with the findings of Egawa et al. that Th-Pok functions late in the specification of the helper T cell lineage (40). Since Kojo et al. had demonstrated repressive regulation of E4m by Th-Pok when the silencer and proximal enhancer were absent (26), we also tested the effect of Th-Pok on CD4 expression in coreNCE/E4m-deficient and -sufficient RLM11 cells. While introduction of Th-Pok in the INT stage cells resulted in small but significant decrease of CD4 on the cell surface of successfully transfected cells, this effect was independent of the presence of coreNCE/E4m, indicating that the Th-Pok effect on CD4 expression may be indirect or that multiple elements may be able to recruit it to the *Cd4* locus.

Interestingly, the enhancer function in the murine CD4SP EL4 cells was barely significant and similar to other studies (29) we did not observe enhancer function in human CD4SP Jurkat cells whether SV-40 promoter or murine *Cd4* promoter was used. One potential explanation for the decrease or lack of *in vitro* enhancer function could be that there is a difference between human and murine transcription factors or binding site requirements, however the highly conserved nature of the element makes us think this is unlikely. Another explanation could be that the enhancer ability is truly INT-stage specific and wanes at the CD4SP stage, where its epigenetic function could be dominant.

Consistent with classical transcriptional enhancer behavior, removing coreNCE/E4m from the *Cd4* locus resulted in a 50% decrease in the rate of CD4 expression and corresponding decreases in the steady state levels of CD4 on the cell surface of INT (RLM11) but not DP (AKR1G1) cell lines (**Figure 3**). Removing coreNCE/E4m from INT stage cells also significantly decreased their ability to upregulate CD4 expression in response to TCR signaling, consistent with the necessary timing for a regulatory element that functions in response to positive selection signals (**Figure 3C**). As the deletion was made in the genome of the cells, it is possible that the previously reported epigenetic function of E4m (41) was also eliminated in the process and what we are observing is not simply loss of expression due to lower canonical enhancer function, but also loss of the demethylation ability of E4m (26). Since cells in culture lose their epigenetic marks and undergo some histone modification reprogramming (42, 43) we think that we are observing primarily the enhancer function in our CRISPR coreNCE/E4m-deleted clones, however further research is needed to rule in or out any E4m-dependent epigenetic contribution.

We observed the NCE-dependency of CD4 expression in post-selection thymocytes *in vivo* as well, where a CD4BAC reporter transgene expresses short-lived EGFP with different kinetics depending on the presence or absence of NCE (**Figure 5**). Although the CD4-reporter EGFP expression levels are low due to the short half-life of the destabilized EGFP protein and low copy number of the transgene, especially in the CD4BACΔNCE-EGFP mice, we can see that EGFP levels increase as cells progress from the signaled DP to the INT stage in the presence but not in the absence of NCE (**Figure 4E**). Since the level of EGFP expression in CD4BACΔNCE-EGFP mouse is at the limit of detection of our flow cytometer, we employed additional methods to determine the effect of NCE on CD4 reporter EGFP expression.

First, we measured steady state mRNA levels of EGFP relative to endogenous *Cd4* in sorted thymocytes populations at each developmental stage, then normalized to the DP stage. There was readily detectable EGFP mRNA in DP thymocytes in both mouse strains, indicating that the transgene was expressed in both mouse strains. Also, it became apparent that NCE-sufficient thymocytes from CD4BAC-EGFP mice significantly increased EGFP expression in the INT stage as compared to DP stage, while in NCE-deficient thymocytes from CD4BACΔNCE-EGFP mice there was a sharp decrease in EGFP mRNA in post-selection thymocytes as compared to DP thymocytes (**Figure 4F**), consistent with the absence of a critical positive regulatory element.

Second, we stabilized the EGFP reporter protein by blocking proteasome degradation and measured the fold change in EGFP fluorescence in proteasome inhibitor treated relative to untreated thymocytes after 24-hour incubation. A fold change bigger than 1 would indicate that both the EGFP molecules already present and newly produced molecules are stabilized, while a fold change equal to 1 would indicate that no additional EGFP molecules were produced for the duration of the treatment. We observed significant accumulation of EGFP at the unsignaled DP stage regardless of the presence of NCE, consistent with our *in vitro* observation that NCE does not function at the DP stage. We think *Cd4* expression at this stage is driven by the proximal enhancer, which is present in both BAC constructs, while the lower accumulation level in the CD4BACΔNCE-EGFP DPs reflects the difference in copy number between the two mouse strains. In contrast, in post selection thymocytes we observed EGFP accumulation only in NCE-sufficient and not in NCE-deficient transgenic mice (**Figure 5**), which together with the sharply decreasing mRNA levels indicates that there is very little new transcription from the *Cd4* gene in the absence of NCE. This outcome is only partially consistent with the 50% decrease in expression we observe *in vitro* and suggest additional function for NCE *in vivo*, presumably via the epigenetic regulation described by others (27, 41).

It is possible that the overall very low levels of EGFP in the CD4BACΔNCE-EGFP mouse are due to the transgene landing in a chromosomal location with a high level of heterochromatin, such as a centromere or a telomere. Insertion location should not affect the *Cd4* locus, as it is known to have an LCR (44), which is present in the BAC used in this study. Another possible explanation for that the lack of EGFP expression in the absence of NCE in post-selection thymocytes could be the diminishing enhancer function of the *Cd4* proximal enhancer, which in the absence of E4m has no other positive element functioning post selection with which to coordinate, and may still be opposed by the silencer until full commitment to the helper lineage is achieved with the expression of Th-Pok (27, 28). Since neither the transcriptional enhancement nor the stabilization of open chromatin marks occur in the absence of NCE *in vivo*, we observed the combined effect as an overall reduction in the EGFP reporter transgene expression.

In conclusion, our study for the first time demonstrates the developmental stage-specific, TCR-responsive, canonical enhancer ability of a highly conserved regulatory element in the *Cd4* locus, coreNCE/E4m, responsible for the CD4 upregulation during positive selection, which is known to ensure error-free commitment to the helper lineage and contributes to the maintenance of CD4 expression in post-selection thymocytes. It would be interesting to explore whether the human equivalent of coreNCE/E4m exhibits both enhancer and epigenetic functions in combination with either of the two human *Cd4* promoters and whether this interaction is necessary for CD4 expression in human macrophages.

## Materials and methods

### Mice

CD4BAC-EGFP and CD4BACΔNCE-EGFP transgenic mice were generated by pronuclear injection of linearized, purified BAC constructs at Duke University and Emory Transgenic Facilities. The transgenic mice were identified by PCR of genomic DNA from toe biopsy samples using the primers SacB.S1 and F8.AS1, specific to the transgene backbone, and the primers CUSScd4p2fw, CUSScd4rv, and 5’EGFP.AS1 to confirm the EGFP insertion (**Supplementary Table S1**). Transgenic founder lines were established by breeding transgene-positive mice to C57Bl/10J mice and monitoring transgene transmission via PCR. The transgene copy number was determined by qPCR (**Supplementary Figure S7**) using TaqMan primers and probes for EGFP (Thermo Fisher #4400291) and *Tfrc* as a copy number reference (Thermo Fisher #4458366). All the mouse strains were housed and bred at the Davidson College Animal Care Facility and handled according to the guidelines set by the Institutional Animal Care and Use Committee.

### Cell culture and treatments

RLM11, a ThPOK-negative CD4^+^CD8^-^ murine thymoma, and AKR1G1, a CD4^+^CD8^+^ murine thymoma (RRID: CVCL_6565), were generously donated by Dr. Bosselut (NIH Laboratory of Immune Cell Biology). EL-4, a CD4^+^CD8^-^ murine T-cell lymphoma (RRID: CVCL_0255), Jurkat, a human CD4+CD8- T-cell leukemia cell line, and VL3-3M2, a CD4^+^CD8^+^ murine thymoma (RRID: CVCL_XF87) were generously donated by Dr. Singer (Experimental Immunology Branch of the NCI). Cell lines and *ex vivo* cells derived from the lymph nodes, spleen, or thymus were incubated at 37°C with 7.5% CO_2_ in CML medium (RPMI 1640 [Fisher 11-875-093] supplemented with 10% charcoal-stripped, heat-inactivated fetal bovine serum [Atlanta Biologicals S11195]; 70 nM 2-β-mercaptoethanol [Sigma-Aldrich 60-24-2]; 0.11 mM nonessential amino acid solution [Atlanta Biologicals B82210]; 0.11 mM sodium pyruvate [Atlanta Biologicals B84010]; and 0.2X penicillin/streptomycin/glutamine solution [Gibco 10378-016]). Cell surface CD4 was removed by following a modified version of the pronase treatment protocol for the study of CD4 and CD8 re-expression in thymocytes (39). Cells were treated with 0.1% pronase (Roche 10165921001) in HBSS for 15 min at 37°C at 5x10^6^ cells/mL, followed by three washes in serum-containing buffer or medium. Stimulation with concanavalin A (Sigma-Aldrich C0412) was performed at 5 µg/mL for RLM11 cells and 10 mg/mL for AKR1G1 cells for 24 hours. Thymocytes from transgenic mice were plated on 24-well plates at a concentration of 5x10^6^ cells/mL with 1 mL of 0 nM, 100 nM, or 1000 nM PS-341 (Selleckchem S1013) in CML media for 24 hours to allow for the accumulation of EGFP. EGFP MFI before and after treatment was measured via flow cytometry.

### Transfection procedure

The day before transfection, the cells were split 1:2 and allowed to grow overnight to a density of 1x106 cells/mL. The cells were spun down at 35×g for 15 minutes at 4°C, washed once with RPMI 1640 without phenol red or L-glutamine, and resuspended at 25x10^6^/mL. For each transfection, 5 μg of a transfection control plasmid (pCD8-CMV or pdsRed-Sensor) and 10 μg of an EGFP reporter plasmid containing combinations of *Cd4* regulatory elements or Th-Pok expression plasmid (pcDNA3Zbtb7b) were added to 5x10^6^ cells (200 μl), incubated at room temperature for up to 30 min, and electroporated using a Harvard Apparatus BTX Gemini Twin wave electroporator in 2 mm gap electroporation cuvettes using a single 285 V, 10 ms square wave pulse. After transfection, 1 mL of prewarmed CML media was added to the sample, which was then transferred to a 6-well plate containing 4 mL of CML, incubated overnight (16-18 hours) for the EGFP reporter plasmids or 48 hours for the Th-Pok expression plasmid, and analyzed via immunostaining and flow cytometry. When performing multiple comparisons to the activity of the promoter alone, a two-tailed t test comparison to a standard value was used to determine significance. For each experiment the promoter alone measurement was used as the standard value and results of the other constructs made relative to it allowing for standardization across trials. Specifically, we calculated a “t-score” by subtracting the standard value from the sample mean, then dividing by the standard error of the mean, and look up this t-score on a T distribution table to find the corresponding probability (p-value) based on the sample size (degrees of freedom) to determine if the difference between the sample and the standard value is statistically significant.

### Immunostaining and flow cytometry

A total of 1x10^6^ of cells were suspended in 100 ml of FACS buffer (HBSS with 0.1% NaN_3_ and 0.1% BSA fraction V) in 5 mL round-bottom tubes, and nonspecific binding was blocked with anti-FcγRIII/II clone 2.4G2 (BD Biosciences Cat# 553140, RRID: AB_394655). Then, the cells were stained for 40 minutes at 4°C with a combination of fluorophore-conjugated antibodies, such as anti-mouse CD4-PE Clone GK1.5 (BD Biosciences Cat# 557308, RRID: AB_396634), anti-human CD3 isotype control [PE-conjugated (BioLegend Cat# 300408, RRID: AB_314062); FITC-conjugated (BioLegend Cat# 300440, RRID: AB_2562046); APC-conjugated (BioLegend Cat# 300439, RRID: AB_2562045)], anti-mouse CD8α-APC (Clone 53-6.7 17-0081-83 eBioscience), anti-mouse TCRβ-Alexa647 (HM3621 Invitrogen), anti-mouse CD69-FITC (Clone H1.2F3 14-0691-81 eBioscience), anti-mouse CD5-FITC (clone 53-7.3), and anti-mouse CD24-PE (Biolegend 101803). When biotinylated antibodies, such as anti-mouse Qa-2-bio (clone 1-1-2 from BD) and anti-mouse TCRβ-bio were used, they were added 15 min before the addition of fluorescent antibodies. Subsequently, the cells were washed twice in FACS buffer, and if biotinylated antibodies were present, the samples were incubated with Streptavidin PerCP-Cyanine5.5 (45-4317-80 eBioscience) for 15 min at room temperature. After another wash, the cells were resuspended in FACS buffer, filtered, and run on an Accuri C6 flow cytometer. The data were analyzed using the Accuri Cflow Plus and FCS Express 4 data analysis software packages. When sorting thymic populations (**Supplementary Figure S8**) for RT-qPCR, cells were resuspended in 1x PBS supplemented with 3% FCS at 20x10^6^/mL, stained as above, resuspended in sorting buffer (1x PBS supplemented with 0.5% FCS), and filtered before being sorted directly into TRIzol-LS. For single cell sorting after CRISPR, no staining was necessary, and the cells were washed, resuspended in sorting buffer and collected in CML media. All sorting was performed on a BD FACSAria II cell sorter.

### RT-qPCR

Total RNA was extracted from sorted populations of thymocytes using TRIzol (Invitrogen #15596026) and a DirectZol RNA Purification Kit (Zymo Research R2060), then converted into cDNA using the Protoscript II First Strand cDNA Synthesis Kit (NEB #E6560S). Rpl13 was used as the internal reference gene, and quantitative PCR was completed using Thermo Scientific’s Luminaris Color HiGreen Low ROX qPCR Master Mix (#K0371) with the primers CD4Ex1F, CD4Ex2R, and EGFP-Reverse (**Supplementary Table S1**) and an Mx3000P real-time PCR machine. For the TaqMan version of the assay, a QS3 real-time PCR machine was used with the Rpl13 assay (Thermo Fisher Mm05910660, #4448489), the TaqMan Fast Advanced Master Mix (Thermo Fisher #4444556), and the primers and probes listed in **Supplementary Table S1**. EGFP expression is presented relative to endogenous *Cd4* expression in the same sample and normalized to the DP population of the same mouse, allowing for comparison of the change of EGFP expression between developmental stages in mice of different transgene copy number.

### CRISPR

To delete the coreNCE/Erm, the CRISPR/Cas9 system was used as follows. The upstream guide (UF2: CACCGAGATAGGGGGCACTCCAAG and UR2: AAACCTTGGAGTGCCCCCTATCTC) and downstream guide (DF5: CACCGCCCCACACCAACGCACGCT and DR5: AAACAGCGTGCGTTGGTGTGGGGC) were cloned separately into the plasmid pSpCas9 BB-2A-GFP PX458, sequence verified, and maxiprepped using the ZymoPURE™ Plasmid Maxiprep Kit (D4202). AKR1G1 or RLM11 cells were transfected with both plasmids using the transient transfection procedure described above. Two days after transfection, single cell sorting of GFP-positive cells was performed for AKR1G1 cells, while limiting dilution was performed for RLM11 cells. Emerging clones were screened using the CheckForward, CheckReverse and DeletionCheck primers (**Supplementary Table S1**). Successful deletion of coreNCE/E4m was confirmed by sequencing.

## Data Availability

The original contributions presented in the study are included in the article/**Supplementary Material**, further inquiries can be directed to the corresponding author/s. The raw data supporting the conclusions of this article will be made available by the authors, without undue reservation.
