# Supplementary material for: A conserved element in the first intron of Cd4 has a lineage specific, TCR signal-responsive, canonical enhancer function that matches the timing of cell surface CD4 upregulation required to prevent lineage choice error
Source: Front Immunol. 2025 Jan 15;15:1469402. doi: 10.3389/fimmu.2024.1469402 (PMC11774700; doi:10.3389/fimmu.2024.1469402)
Supplement: Supplementary file 1 [file DataSheet1.pdf]

frontiers

Supporting Information

Table of Contents

Supporting Figures	page
**Figure S1.** A sensitive GFP reporter assay to detect differences in CD4 cis-element function.	2
**Figure S2.** NCE does not function in the human CD4SP cell line Jurkat	3
**Figure S3.** Effect of Th-Pok transfection into RLM11 and AKR1G1 cells in the presence or absence of coreNCE/E4m	4
**Figure S4.** Bacterial Artificial Chromosome clone bMQ391f08.	5
**Figure S5.** Deletion of NCE by BAC recombineering.	6-7
**Figure S6.** Insertion of EGFP by BAC recombineering.	8
**Figure S7.** Verification of recombineered BACs by PFGE.	9
**Figure S8.** Screening of founder CD4BAC-EGFP and CD4BACΔNCE-EGFP mice.	10
**Figure S9.** Relative copy number of BAC transgenes in CD4BACΔNCE-EGFP and CD4BAC-EGFP mice.	11
**Figure S10.** Gating strategy for sorting thymocyte subpopulations.	12
**Supporting Tables**	
**Table S1.** List of primers and probes used in this study.	13
**Table S2.** Two-way ANOVA analysis of Th-Pok transfected RLM11 cells +/- coreNCE/E4m.	14
**Supporting Methods**	
**1.1 BAC Recombineering**	15-16
**1.2 Plasmids and reporter constructs**	16-18

Supporting Figures

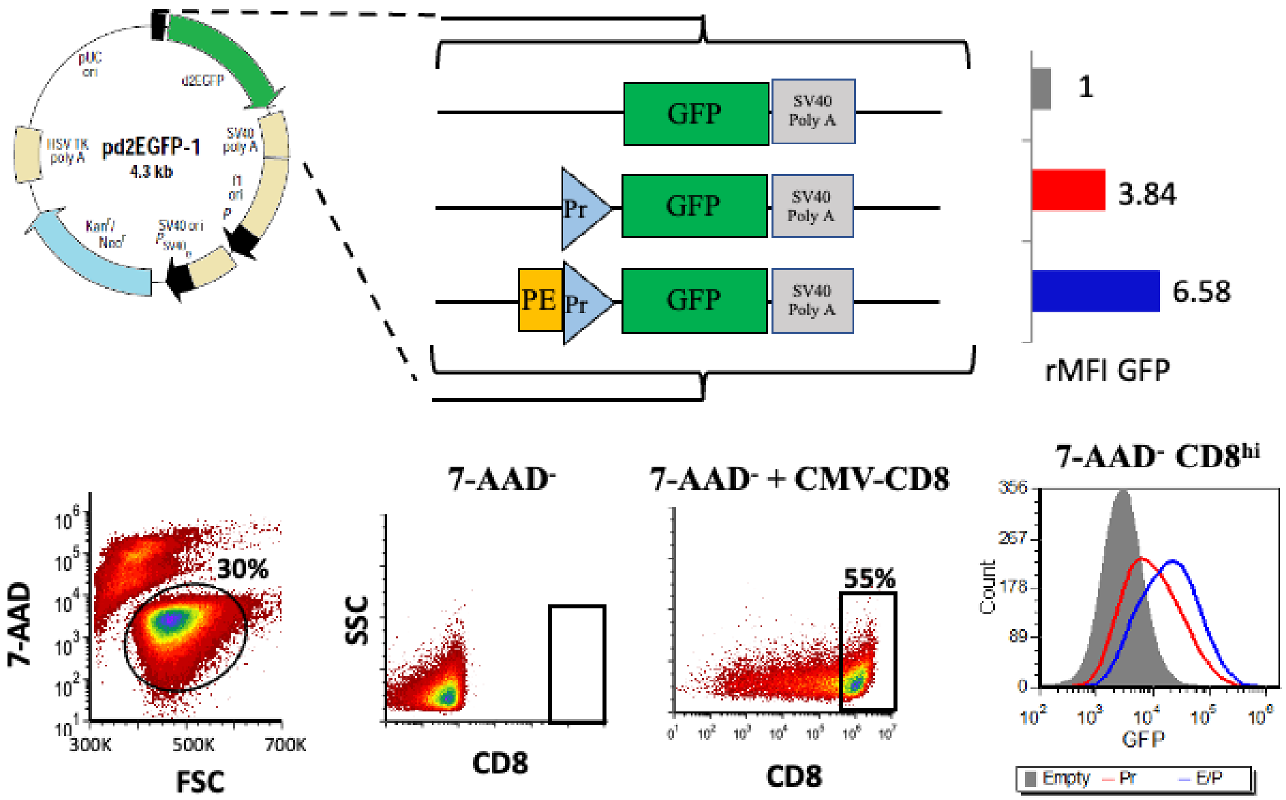

Supplementary Figure 1. A sensitive GFP reporter assay to detect differences in CD4 cis-element function. Schematic representation of the pd2EGFP-1 reporter plasmid with expanded section where cis-regulatory elements Pr (Cd4 promoter) and PE (Cd4 proximal enhancer) were introduced. After overnight incubation of RLM11 cells transfected with reporter plasmid using a single 285 V, 10 ms square wave pulse, cells were immunostained and analyzed by flow cytometry. Surviving cells were identified as 7AAD$^{neg.}$, large cells (left FACS plot, elliptical gate). CD8 expression in the absence (middle FACS plot) or presence (right FACS plot) of CMV-CD8 (transfection control plasmid) co-transfection was used to identify the highly transfected RLM11 cells as the CD8^{hi} population (black rectangle) throughout the study. When CD8+ cells were transfected, pdsRed plasmid was used as a transfection control instead. Histogram represents GFP level of expression of the CD8^{hi} RLM11population transfected with pd2eGFP-1 “empty” vector (grey), d2eGFP-1.Pr (Pr, red) and pd2eGFP-1.PE.Pr (E/P, blue). Bar graph represents the average of 4 trials. Using a two-tailed T-test comparison to a standard value, with the empty vector set to the standard value of 1.0, the rMFI of the Pr construct is significantly different than the rMFI of the E/P construct, $p = 0.032$, indicating that this transient transfection assay is sensitive enough to detect differences in GFP fluorescence due to different cis-regulatory elements’ function.

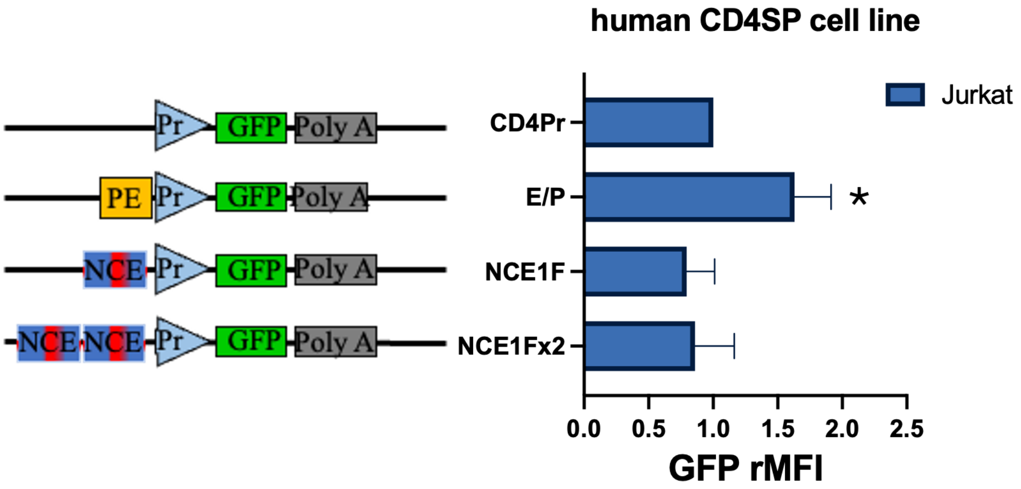

Supplementary Figure 2. NCE does not function in the human CD4SP cell line Jurkat.

Schematic representations of the constructs tested are shown on the left of each panel. Pr = *Cd4* promoter; PE = *Cd4* proximal enhancer; NCE = novel cis-acting element; red bar within NCE = 150 bp highly conserved sequence from Figure 1 (coreNCE/E4m). Bar graphs represent the relative EGFP MFI of successfully transfected cells, identified as in Figure S1, after transient transfections of each construct together with a transfection control plasmid CD8α-CMV in Jurkat cells, as measured using an AccuriC6 flow cytometer and Cflow Plus Software. Significant enhancement of *Cd4* promoter-driven EGFP expression was determined using a two-tailed t test comparison to a standard value; * indicates $p < 0.05$, n=3. Error bars represent the standard error of the mean.

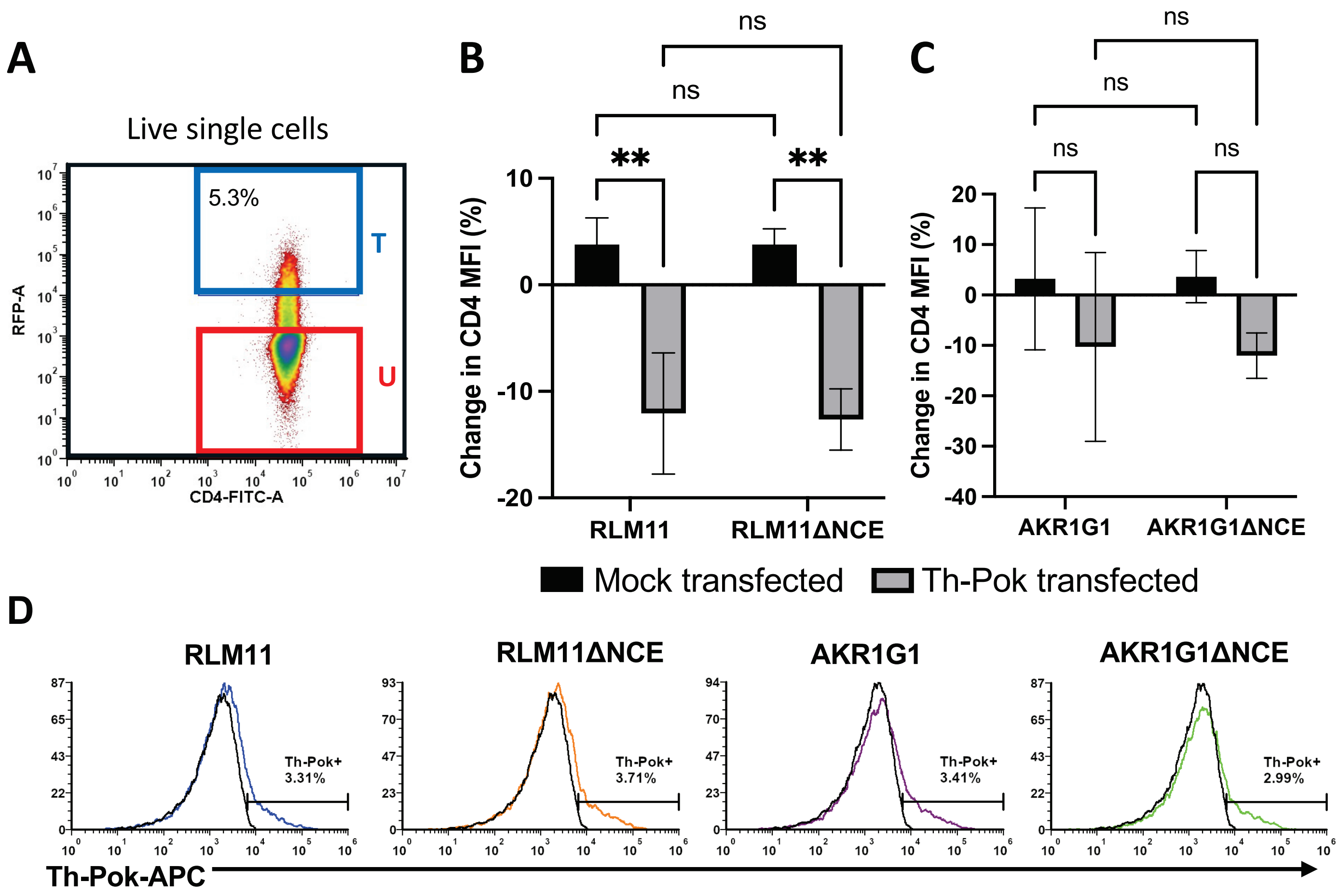

RLM11 and AKR1G1 cell lines.

A. A representative FACS plot of RLM11 cells transfected with the reporter pRed and immunostained for CD4. The highly transfected (RFP^{hi}, blue box, T) and untransfected (RFP-; red box, U) populations were used to calculate the percent change in CD4 MFI between the two populations according to the formula % change = [(T-U)/U]*100. Percent change in CD4 MFI following mock transfection with pRed alone (black) or in combination with a Th-Pok expression vector (grey) of coreNCE/E4m sufficient or deficient RLM11 **(B)** and AKR1G1 **(C)** cells. Bar graph represents the average of three trials. Statistical significance was determined using two-way ANOVA with the Tukey HSD post-hoc test (Table S2). **D.** Histograms represent internal immunostaining followed by flowcytometry for Th-Pok in Th-Pok-transfected (color histogram) or mock-transfected (black histogram) in the indicated cells fixed and permeabilized 48h after transfection.

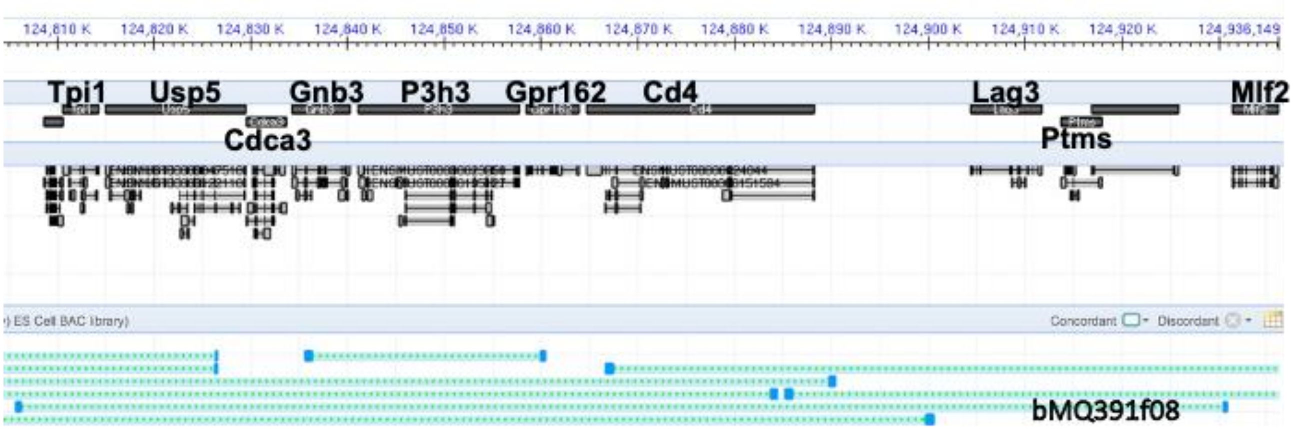

Supplementary Figure 4. Alignment of the 125kb genomic DNA insert of the Bacterial Artificial Chromosome clone bMQ391f08 used in this study to the Cd4 locus on mouse Chromosome 6, encompassing all known Cd4 gene regulatory elements.

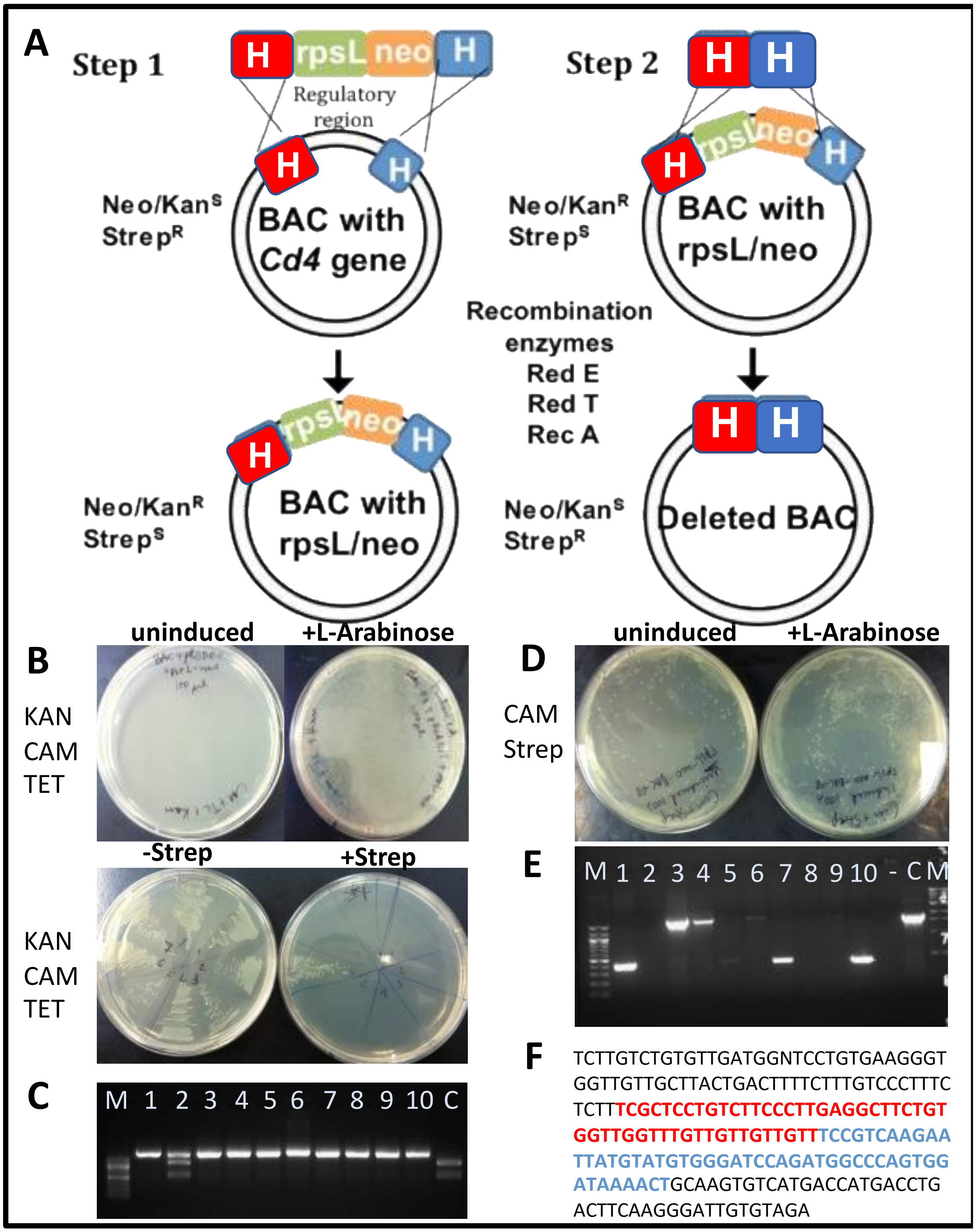

Supplementary Figure 5. Deletion of NCE by BAC recombineering A) Schematic representation of the selection/counter-selection strategy for NCE deletion. Step 1, selection, replaces NCE with the linear rpsL/neo cassette, flanked by homology arms to the boundaries of NCE, by homologous recombination using the enzymes RedE and RedT, which are delivered to the bacteria on a separate plasmid. Bacteria containing BAC with successful recombination are Kan^R due to the Neo gene and Strep^S due to the rpsL gene and clones are verified by colony PCR. Step 2, counter-selection, deletes the rpsL/neo cassette by homologous recombination with linear DNA that consists of the two homology arms. Counter-selection with Streptomycin reveals the deleted BAC, which is confirmed

by colony PCR. B) Expression of the recombinase proteins was induced in the presence of Arabinose and the rpsL/neo cassette was introduced by electroporation. BAC with successful recombination events support bacterial growth in the presence of Kanamycin but are Streptomycin sensitive. C) The successful insertion of the selection cassette was confirmed by PCR and loss of the SpeI restriction site when NCE is deleted. D) Recombinase protein expression was induced with Arabinose again and 100bp DNA sequence consisting of the two homology arms was introduced by electroporation. BAC with successful replacement of the selection cassette by the homology arms DNA support bacterial growth in the presence of Streptomycin. E) Colony PCR of Streptomycin resistant clones 1.6 kb band indicates absence of deletion; 400 bp band indicates successful deletion. C = unmodified CD4BAC; M = 100bp ladder. F) 50% of the colonies from the induced plate contained a seamless deletion of NCE between the homology arms. Left homology arm sequence is in red; right homology arm sequence is in blue.

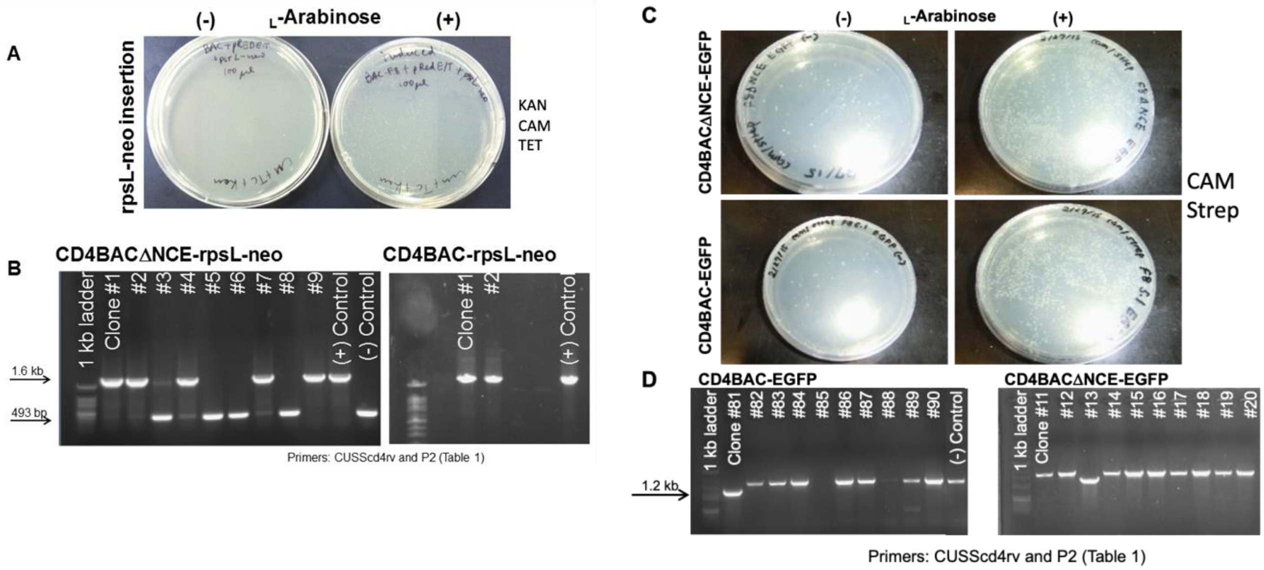

Supplementary Figure 6. Insertion of EGFP by BAC recombineering. The same selection/counter-selection strategy as in Figure S3 was used for EGFP insertion, except Step 1 replaces 80bp of Exon 2 by homologous recombination using the enzymes RedE and RedT and the rpsL/neo cassette flanked by homology arms corresponding to the ends of the targeted sequence, and Step 2 replaces the rpsL/neo cassette by homologous recombination with linear DNA that consists of EGFP flanked by the same two homology arms. In both steps recombinases were induced with L-Arabinose and the antibiotic selection/counter-selection scheme was the same as in Figure S3. A. Expression of the recombinase proteins was induced in the presence of Arabinose and the rpsL/neo cassette was introduced by electroporation. BAC with successful recombination events support bacterial growth in the presence of Kanamycin but are Streptomycin sensitive. B. Electrophoresis images of colony PCR products showing the insertion of the rpsL/neo cassette as a 1.6 kb band. C. L-Arabinose-induced groups after Step 2 have a higher rate of survival compared to non-induced groups in the presence of Streptomycin. Any false positive colonies without the egfp insertion could have resulted from recombination within the rpsL-neo cassette, conferring streptomycin resistance. D. Electrophoresis images of colony PCR products showing successful replacement of the rpsL-neo cassette, resulting in a smaller band of 1.2 kb. Clones #81 (CD4BAC-EGFP) and #13 (CD4BACDNCE-EGFP) were chosen for the subsequent purification procedure and generation of BAC-transgenic mice.

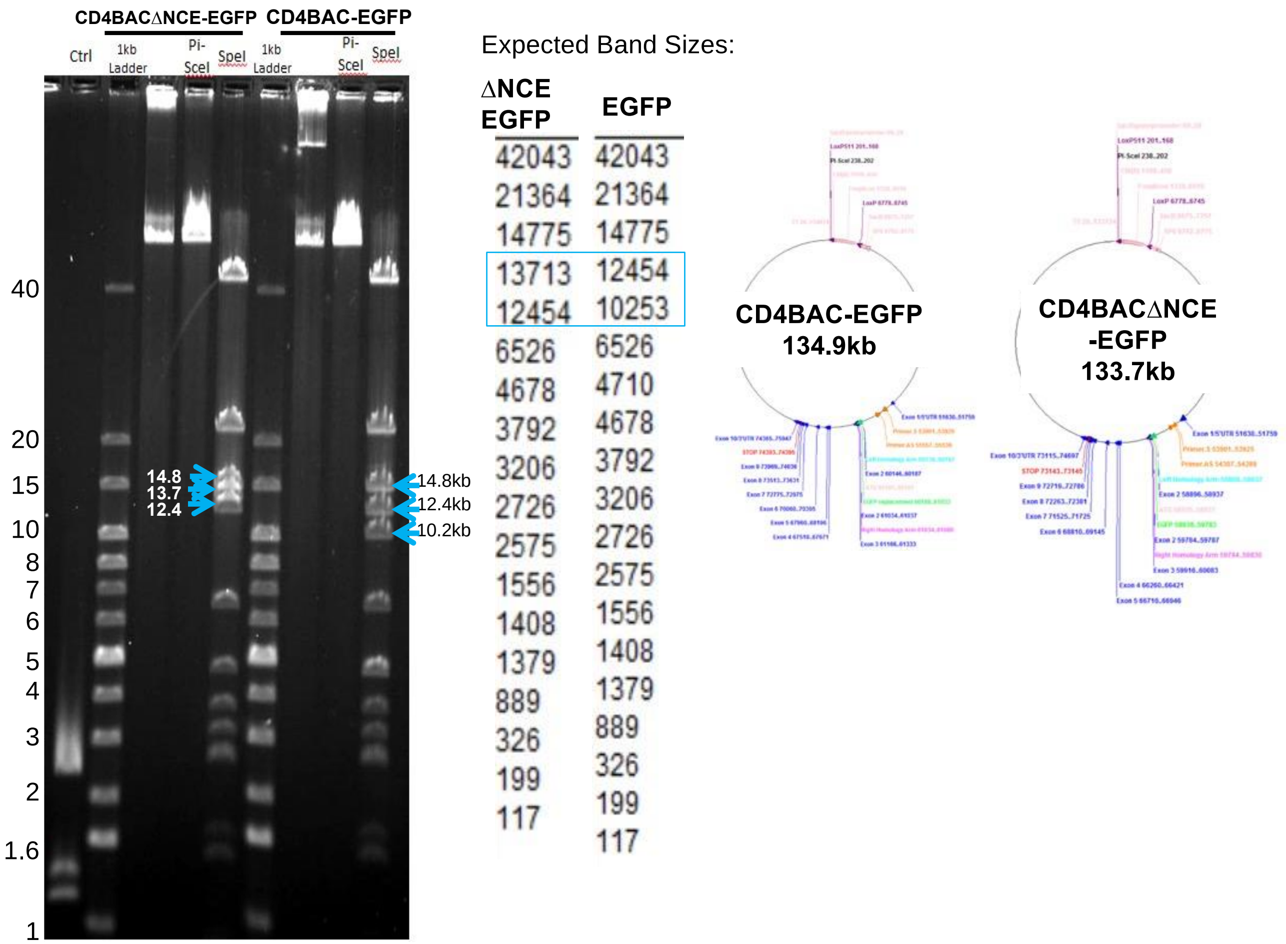

Supplementary Figure 7. Verification of recombineered BACs by PFGE. Pulsed field gel electrophoresis of SpeI digested CD4BAC-EGFP shows a 10.2kb band demonstrating successful insertion of EGFP. The 13.7kb band in CD4BACΔNCE-EGFP shows successful insertion of the EGFP and absence of NCE. Digestion with PI-SceI shows successful linearization for pronuclear injection.

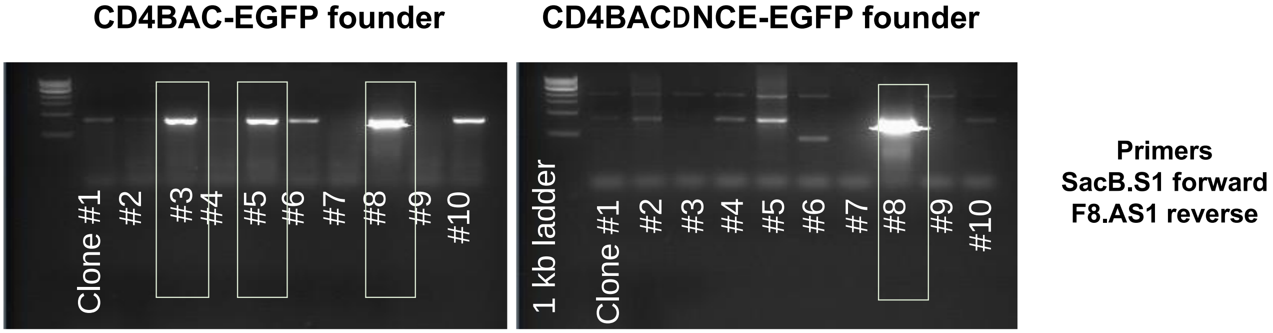

Supplementary Figure 8. Screening of founder CD4BAC-EGFP and CD4BACΔNCE-EGFP mice. Three strongly positive mice with CD4BAC-EGFP and one strongly positive mouse with the CD4BACΔNCE-EGFP were identified by PCR of genomic DNA and gel electrophoresis and used to start BAC-transgenic lines by breeding to C57BL/10 mice. Out of three founder lines for CD4BAC-EGFP, #3 failed to produce progeny, #5 did not transmit the transgene, and #8 had germline transmission. Only one founder line was generated for CD4BACDNCE-EGFP, and it had germline transmission of the transgene.

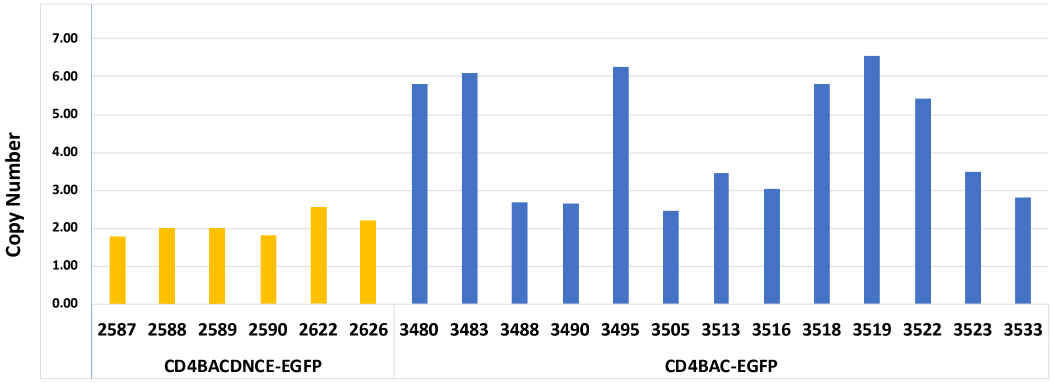

Supplementary Figure 9. Relative copy number of BAC transgenes in CD4BACΔNCE-EGFP and CD4BAC-EGFP mice. Genomic DNA samples from CD4BACΔNCE-EGFP and CD4BAC-EGFP mice were analyzed by duplex qPCR using TaqMan Copy Number Reference Assay for a housekeeping gene, Tfcr, known to be two copies per genome (ThermoFisher #4458366) and an EGFP TaqMan Copy Number Variance Assay (ThermoFisher Mr00660654_cn, #4400291) TaqMan Fast Advanced Master Mix (ThermoFisher #4444556). The CD4BACΔNCE-EGFP strain has stabilized at transgene copy number of 2, while the CD4BAC-EGFP strain is not homozygous yet, with heterozygous mice having transgene copy number of 3 and homozygous mice with copy number of 6.

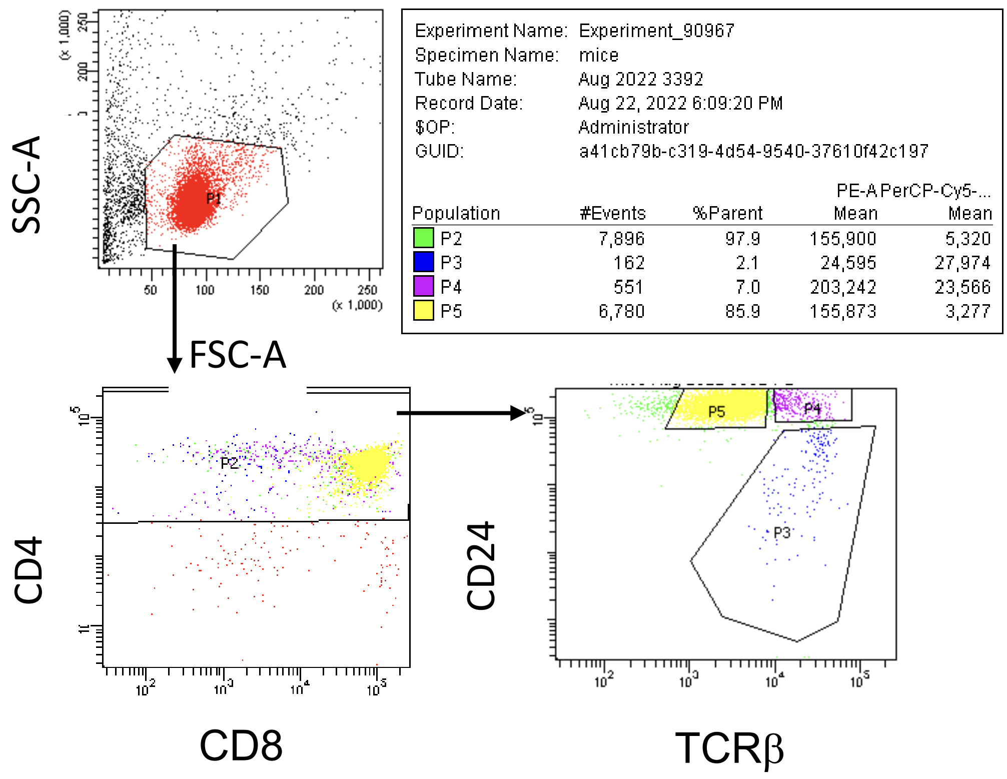

Supplementary Figure 10. Gating strategy for sorting thymocyte subpopulations. Live thymocytes (P1) were analyzed for CD4 and CD8 expression and the CD4+ cells (P2) further analyzed for CD24 and TCRb expression to separate double positive (P5), intermediate (P4) and CD4 single positive (P3) populations. The location of the gated cells in P3, P4, and P5 is indicated on the CD4/CD8 plot by back-gating in the corresponding gate color.

Table S1. List of primers and probes used in this study.

Primer name	Sequence	Description
GS1.2F	AGTCTCCTTCATTCCTATGCCTCTCC	PCR of NCE
GS1.2R	TGCCTGTTGGTCCTTGTCC	PCR of NCE
PrForward	GGATCCGTCATCTCTAGAACAGCC	CD4 promoter-specific with BamHI site
PrReverse	AGATCTGAGGGCTCGAGACTTTGC	CD4 promoter-specific with BglII site
PEnForward	GTCGACTTGGGGTTCAAATTTGAGC	CD4 proximal enhancer-specific with SalI
NCEF5forward	ACTAGTTGCAGCTCAGAGGAAGACATCC	core NCE with SpeI site
NCEF5reverse	ACTAGTTGGAGGAACGAGGGCATTCC	core NCE with SpeI site
SacB. S1	ATGTACCTGGTTTGAAGTGATCAG	BAC-specific
F8. AS1	ACTATAGTCTAGTGGTAGGGCATG	BAC-specific
CUSScd4p2fw	TCTTAGTTTGGCAGGACCTTTGGG	Cd4 gene-specific (intron1)
CUSScd4RV	TCCCTTGAGTGACAGCTAGGAGTT	Cd4 gene-specific (exon 3)
5'EGFP. AS1	ATGGCGGACTTGAAGAAGTCGTGC	Screening for EFGP containing BAC
CheckForward	TGGGTGGCATCTGAGGAGAG	Screening CRISPR clones
CheckReverse	GGAAAACCTTGATGAGCGGG	Screening CRISPR clones
DeletionCheck	AGGCTGAGGATGTGGGAATC	Screening CRISPR clones
CD4Ex1F	GAAGCAGAGTGAAGGAAGGACTG	qPCR for endogenous CD4, transgene and TaqMan multiplex assay
CD4Ex2R	CTTAAGAGAGATGGCTCGGC	qPCR for endogenous CD4
EGFP-Reverse	CAGGGTCAGCTTGCCGTAG	qPCR for CD4BAC-EGFP
TaqMan EGFP probe	TGAGCAAGGGCGAGGAGCTGTT	ABY with a QSY quencher
TaqMan Cd4 probe	TGCCGAGCCATCTCTCTTAGGCG	FAM with MGBNFQ quencher
EGFPqPCR	TCCAGCTCGACCAGATG	Reverse primer for TaqMan multiplexing
CD4qPCR	GCAGCAGCAGCAGCAAG	Reverse primer for TaqMan multiplexing

deltaNCE rpsL/neoF with a homology arm for BAC recombineering
TCGCTCCTGTCTTCCCTTGAGGCTTCTGTGGTTGGTTTGTTGTTGTTGTTggcctggtgatgatggcgggatcg

deltaNCE rpsL/neoR with a homology arm for BAC recombineering
AGTTTTATCCACTGGGCCATCTGGATCCCACATACATAATTCTTGACGGAtcagaagaactcgtcaagaaggcg

Ex2 rpsL/neoF with a homology arm for BAC recombineering
TTTTGTAGGCTCAGATTCCCAACCAACAAGAGCTCAAGGAGACCACCATGggcctggtgatgatggcgggatcg

Ex2 rpsL/neoR with a homology arm for BAC recombineering
TCCTTGCCCCATCTGCCACTGGTCCCCAAGTCAGAAAACTTACACAGtcagaagaactcgtcaagaaggcg

Ex2 EGFP-F with a homology arm for BAC recombineering
TTTTGTAGGCTCAGATTCCCAACCAACAAGAGCTCAAGGAGACCACCATGgtgagcaagggcgaggagc

Ex2 EGFP-R with a homology arm for BAC recombineering
TCCTTGCCCCATCTGCCACTGGTCCCCAAGTCAGAAAACTTACACAGgcatctacacattgatcctagc

Table S2. Two-way ANOVA analysis of Th-Pok transfected RLM11 cells +/- coreNCE/E4m

ANOVA table	SS	DF	MS	F (DFn, DFd)	P value
Interaction	0.2370	1	0.2370	F (1, 8) = 0.01933	P=0.8929
+/- coreNCE/E4m	0.2343	1	0.2343	F (1, 8) = 0.01911	P=0.8935
+/- Th-Pok	782.6	1	782.6	F (1, 8) = 63.85	P<0.0001
Residual	98.05	8	12.26		

1.1 BAC recombineering

The BAC clone bMQ391f08 was purchased from Source BioScience and contains 125 kb of genomic DNA from chromosome 6, including the *Cd4* gene locus (Figure S2). To delete 1.25kb of NCE and insert destabilized EGFP (dsEGFP) immediately downstream of the start codon of the *Cd4* gene, BAC recombineering was performed using the Selection/Counterselection BAC Modification Kit by Gene Bridges (1) following the manufacturer's protocol (Fig. S5A). Briefly, all steps involving electroporation of *E. coli* cells were performed at 1350 V, 10 µF, and 600 Ohms using 1mm cuvettes and a Gemini SC Electroporation Generator (45-2001 BTX Harvard Apparatus). Following electroporation, the cells were incubated at 37°C for 2 hours with shaking to optimize cell survival prior to selective plating. LB medium for colony expansion and plate selection was supplemented as necessary with combinations of chloramphenicol, tetracycline, kanamycin, or streptomycin at concentrations of 15, 3, 50, and 50 µg/mL, respectively; 10% L-arabinose (A-3256 Sigma) was used for the pRedE/T induction step. The rpsL-neo cassettes for NCE deletion and EGFP insertion and the EGFP cassette with homology arms were generated by PCR using the primer pairs deltaNCE rpsL/neoF and deltaNCE rpsL/neoR, Ex2 rpsL/neo-F and Ex2 rpsL/neo-R, and Ex2 EGFP-F and Ex2 eGFP-R respectively (Table S1). The linear DNA containing only NCE homology arms for NCE deletion was synthesized as two complementary 100 bp oligos that were then annealed. All cassettes were gel purified using the Zymoclean Gel DNA Recovery Kit (Cat# D4001). PCR templates for screening BAC clones were prepared by boiling bacterial colonies at 98°C for 5 minutes. The elongation temperature for the PCR was set to 59.5°C for the CUSScd4rv and CUSScd4p2fw primer combination and 58°C for the GS1.2F and GS1.2R primer combination. A shift in the PCR product size from 1.6 kb to 0.4 kb indicated successful

deletion of NCE (Figure 5E). A shift in the PCR product size from 1.6 kb to 1.2 kb indicated a successful replacement of the rpsL/neo cassette with the EGFP cassette (Figure S6D clones 81 and 13). The diagnostic results and PFGE of both constructs are shown in Figure S7.

1.2 Plasmids and reporter constructs

The following plasmids were used as control plasmids without modifications: pdsRed-Sensor, a short half-life red fluorescent protein variant expression vector driven by the cytomegalovirus promoter and enhancer (Addgene plasmid: 22743), and pCD8α-CMV, an expression vector containing a truncated murine CD8α gene lacking the cytosolic tail of the CD8α protein driven by the heterologous cytomegalovirus promoter-enhancer combination (2,3), generously donated by Dr. Bosselut at the NIH Laboratory of Immune Cell Biology. The pd2EGFP-1 vector with a destabilized enhanced green fluorescent protein variant that lacks positive regulatory elements (Addgene Plasmid: 21280) was modified by the addition of a variety of *Cd4* regulatory elements, as described below.

The known *Cd4* promoter (550 bp) (Pr), the 1.2 kb interest region (NCE), and the 300 bp conserved core region of NCE (Frag5) were amplified from genomic DNA of the inbred mouse strain C57BL/6J by PCR using the primers listed in Table S1 and inserted into pCR™4-TOPO using the TOPO® TA Cloning Kit (Invitrogen K4575-02). When necessary, the inserts and vectors were gel purified using the Zymoclean™ Gel DNA Recovery Kit (Zymo D4001). When appropriate, the vectors were dephosphorylated with calf intestinal phosphatase (NEB # M0290S) or Antarctic phosphatase (NEB #M0289S) before ligation using a Quick Ligation Kit (NEB #M2200S). All restriction endonucleases were purchased from NEB. A *Cd4* proximal enhancer fused to the *Cd4* promoter (640 bp) was amplified from

the GFP reporter vector p4-e/p-eGFP-sil, kindly donated by Dr. Bosselut. After cloning, the PCR products were transferred to the pd2EGFP reporter plasmid. The orientation and insertion number were confirmed by restriction enzyme digestion or sequencing as follows [full name followed by (abbreviated name)]:

pd2EGFP-1.Pr (Pr) contains the C*d4* promoter ligated into the BamH-I site of pd2EGFP-1 upstream of EGFP. Single insertion and correct orientation were confirmed by BamH-I digestion. pd2EGFP-1.En.Pr (E/P) contains the C*d4* proximal Enhancer/Promoter ligated into Sal-I/BamH-I-digested pd2EGFP-1. Single insertion was confirmed with a Sal-I digest. **pd2EGFP-1.NCE1F.Pr** (NCE1F) and **pd2EGFP-1.NCE1F.NCE1F.Pr** (NCE1Fx2) contain the 1.2 kb NCE fragment ligated once or twice, respectively, into the EcoR-I site of pd2EGFP-1.Pr upstream of the *Cd4* promoter. A single insertion was confirmed in NCE1F by linearization with Spe-I, and the correct orientation was confirmed using a Bgl-II digest that generated two bands 350 bp and 6.2 kb in size. A double insertion of NCE in NCE1Fx2 was confirmed using a Spe-I digest, which generated a 6.5 kb band and a 1.2 kb band. Orientation was confirmed using a Bgl-II digest, generating three bands at 6.2 kb, 1.2 kb, and 350 bp. The **pd2EGFP-1.Pr.NCE2F** (NCE2F) and **pd2eGFP-1.Pr.NCE2R** (NCE2R) contain the 1.2 kb NCE inserted into pd2EGFP-1.Pr’s Afl-II site downstream of EGFP. A single insertion was confirmed for both plasmids using a Spe-I digest. The orientation was confirmed using sequencing. **pd2EGFP-1.NCE1F5.Pr** (Frag5) has a 300 bp fragment containing the 150 bp highly conserved coreNCE inserted into the EcoRI site of pd2EGFP-1.Pr plasmid upstream of the *Cd4* promoter. The **pd2EGFP-1.NCE1F5.Pr.NCE2F5** (Frag5x2) has a second Frag 5 inserted into the Afl-II site of pd2EGFP-1.NCE1F5.Pr downstream of EGFP.

1. Muyrers JPP, Zhang Y, Stewart AF. Techniques: Recombinogenic engineering–new options for cloning and manipulating DNA. *Trends in Biochemical Sciences* (2001) 26:325–331. doi: 10.1016/S0968-0004(00)01757-6

2. Bosselut R, Zhang W, Ashe JM, Kopacz JL, Samelson LE, Singer A. Association of the adaptor molecule LAT with CD4 and CD8 coreceptors identifies a new coreceptor function in T cell receptor signal transduction. *JExpMed* (1999) 190:1517–1526.

3. Zamoyska R, Derham P, Gorman SD, von Hoegen P, Bolen JB, Veillette A, Parnes JR. Inability of CD8α′polypeptides to associate with p56lck correlates with impaired function in vitro and lack of expression in vivo. *Nature* (1989) 342:278–281. doi: 10.1038/342278a0
